# Steering the product spectrum in high-pressure anaerobic processes: CO_2_ partial pressure as a novel tool in biorefinery concepts

**DOI:** 10.1186/s13068-023-02262-x

**Published:** 2023-02-18

**Authors:** Pamela Ceron-Chafla, Jo de Vrieze, Korneel Rabaey, Jules B. van Lier, Ralph E. F. Lindeboom

**Affiliations:** 1 Sanitary Engineering Section, Department of Water Management, Delft University of Technology, Stevinweg 1, 2628 CN Delft, The Netherlands; 2 Center for Microbial Ecology and Technology (CMET), Ghent University, Coupure Links 653, 9000 Ghent, Belgium; 3 Center for Advanced Process Technology for Urban Resource Recovery (CAPTURE), Coupure Links 653, 9000 Ghent, Belgium

**Keywords:** Elevated pCO_2_, High-pressure carboxylates production, Veillonellaceae, Succinate

## Abstract

**Background:**

Elevated CO_2_ partial pressure (pCO_2_) has been proposed as a potential steering parameter for selective carboxylate production in mixed culture fermentation. It is anticipated that intermediate product spectrum and production rates, as well as changes in the microbial community, are (in)directly influenced by elevated pCO_2_. However, it remains unclear how pCO_2_ interacts with other operational conditions, namely substrate specificity, substrate-to-biomass (S/X) ratio and the presence of an additional electron donor, and what effect pCO_2_ has on the exact composition of fermentation products. Here, we investigated possible steering effects of elevated pCO_2_ combined with (1) mixed substrate (glycerol/glucose) provision; (2) subsequent increments in substrate concentration to increase the S/X ratio; and (3) formate as an additional electron donor.

**Results:**

Metabolite predominance, e.g., propionate vs. butyrate/acetate, and cell density, depended on interaction effects between pCO_2_–S/X ratio and pCO_2_–formate. Individual substrate consumption rates were negatively impacted by the interaction effect between pCO_2_–S/X ratio and were not re-established after lowering the S/X ratio and adding formate. The product spectrum was influenced by the microbial community composition, which in turn, was modified by substrate type and the interaction effect between pCO_2_–formate. High propionate and butyrate levels strongly correlated with Negativicutes and Clostridia predominance, respectively. After subsequent pressurized fermentation phases, the interaction effect between pCO_2_–formate enabled a shift from propionate towards succinate production when mixed substrate was provided.

**Conclusions:**

Overall, interaction effects between elevated pCO_2_, substrate specificity, high S/X ratio and availability of reducing equivalents from formate, rather than an isolated pCO_2_ effect, modified the proportionality of propionate, butyrate and acetate in pressurized mixed substrate fermentations at the expense of reduced consumption rates and increased lag-phases. The interaction effect between elevated pCO_2_ and formate was beneficial for succinate production and biomass growth with a glycerol/glucose mixture as the substrate. The positive effect may be attributed to the availability of extra reducing equivalents, likely enhanced carbon fixating activity and hindered propionate conversion due to increased concentration of undissociated carboxylic acids.

**Supplementary Information:**

The online version contains supplementary material available at 10.1186/s13068-023-02262-x.

## Background

The emergence of the biorefinery concept, where fuel and chemicals production from (waste) biomass feedstock are envisioned [1], has become a strong driver for product spectrum diversification in anaerobic processes. It has motivated the inclusion of carboxylates production, such as propionate and butyrate and bulk chemicals, such as succinate, in addition to biogas from anaerobic digestion (AD), as (bio)products of interest [2–4]. Anaerobic processes using complex substrates rely on trophic diversity and interspecies interactions to carry out the required bioconversions; thus, the formation of specific intermediates and final products in open microbiomes depends on prevailing operational conditions [5–7]. Due to the interconnection between operational conditions, the intermediate products profile, microbial community and productivity/selectivity, management strategies have been proposed to boost performance by manipulating process parameters (operational-based strategy) and through biomass acclimation and bioaugmentation (microbial-based strategy) [8].

Changes in substrate concentration [9–11], pH [12–14] and temperature [15, 16] are known to influence the profile of intermediate products. Reactor operation [17, 18], substrate-to-biomass (S/X) ratio [19, 20] and headspace composition [21–23] can also modify the intermediate product spectrum. However, thus far, no successful strategies for targeted carboxylates production as intermediate products have been reported. High-pressure anaerobic digestion (HPAD) is considered an innovative technology with potential for direct biogas upgrading [24, 27]. In an HPAD reactor, the partial pressure of biogas components, i.e., CO_2_, may play a role in pathway steering and selectivity in intermediate product formation [25, 26]. The latter, thus far, remains a limitedly studied process due to its operational complexity and high capital expenditure. Nonetheless, possible changes in the community composition, pathways up- or down-regulation, possible pressure-induced alterations of enzymatic activity in HPAD, may have a decisive role in intermediate (product) formation.

An increased CO_2_ partial pressure (pCO_2_) in HPAD reactors may result from an autogenerated build-up in operational pressure [26]. Previous work showed that bioprocesses operating at high pCO_2_ experienced toxicity and acidification effects [26]. Additionally, high pCO_2_ impaired substrate transport over the microbial cell membrane, due to a decreased membrane potential [28] and imposed kinetic, bio-energetic and physiological limitations [29]. Using a pressure-adapted inoculum, authors observed that low methane production rates and propionate accumulation correlated with increasing pCO_2_, constituting pioneering evidence of a potential steering role of elevated pCO_2_ in HPAD [26]. Intermediate product formation does not only depend on the (re-) distribution of organic carbon from the original substrate and the possible role of pCO_2_ on the thermodynamics of (de)carboxylation reactions, but also on the availability of reducing equivalents and the ratio NADH/NAD^+^ [30, 31].

At the macro-process level, changes in the degree of reduction of the employed substrate [32, 33], high S/X ratio or provision of an additional electron donor [34] can be applied to alter the availability and the flux of reducing equivalents. However, an increasing S/X ratio may impair bioconversions in non-adapted biomass, due to kinetic limitations in the production and utilization of intermediates [35]. Substrate specificity and biomass adaptation at increasing substrate concentrations has been pivotal in selecting a microbial community that is more resilient to the detrimental effects of elevated pCO_2_ [36] and, most likely, also fluctuations in the S/X ratio.

Under the premise that microbial resiliency is attained, elevated pCO_2_ could play a role in steering metabolic pathways, because of its tuning effect in enzyme activities related to (de)carboxylation of intermediates [37–39]. These reactions are relevant for the breakdown of substrates sharing the glycolytic pathway, e.g., glucose and glycerol, where carbon atoms and electrons are distributed towards the reductive (propionate) or oxidative (acetate) branch of the pathway in response to growth conditions [40]. Elevated pCO_2_ could also modify the intermediate product spectrum via autotrophic CO_2_ fixation. Acetogenic bacteria, such as *Clostridium* spp., which are crucial in anaerobic microbiomes, can fix CO_2_ into acetyl-CoA via the Wood–Ljungdahl pathway (WLP), provided the availability of reducing equivalents [41]. Mixotrophic acetogenic metabolism, i.e., concurrent heterotrophic and autotrophic growth with high acetate production [42–44], can be enhanced if sugars and CO_2_ are present. Increasing acetate concentrations can favor chain elongation processes with lactate or ethanol [10, 45], as long as acetotrophic methanogenic activity is inhibited [46].

Elevated pCO_2_ could also cause shifts in the microbial community structure, indirectly impacting the intermediate product spectrum. As an environmental driver, elevated pCO_2_ could select carbon fixation traits, leading to a predominance of specific acetogens, such as *Clostridium* spp. [47]. Combined pCO_2_–pH effects may favor the predominance of acid-resistant groups from the phyla Chloroflexi and Firmicutes [48]. Depending on the electron transfer mediator (H_2_, formate), elevated pCO_2_ may also restructure the methanogenic community [49]. The interplay of pCO_2_ and substrate concentration impacting the S/X ratio could modify syntrophic interactions [50], and may cause metabolic uncoupling [51], potentially affecting the intermediate product spectrum.

Overall, there is ample evidence that elevated pCO_2_ influences anaerobic processes in multiple ways. However, the role of pCO_2_ is insufficiently understood to use it as a steering parameter for specific carboxylate production in HPAD. It remains unclear how elevated pCO_2_ could interact with process conditions to ultimately target a particular product, e.g., succinate. In this work, interaction effects of elevated pCO_2_ with (a) the provision of mixed substrate (glycerol/glucose), (b) high substrate concentration increasing the S/X ratio and (c) the presence of an additional electron donor (formate) were investigated. The mixed substrate was provided on the grounds of substrate divergence to propionate production via the succinate pathway (glycerol) and ATP provision to satisfy maintenance and growth requirements (glucose) [52, 53]. A high S/X ratio was imposed as a selection mechanism to favor fermentative and suppress methanogenic activity, due to metabolic uncoupling. Finally, we assessed the effects of formate addition, concomitant to elevated pCO_2_, to stimulate carbon fixating activity and likely promote the formation of reduced intermediates (e.g., succinate), due to the increased availability of reducing equivalents.

## Results

### Mixed substrate conversion in batch operation under elevated pCO_2_

#### Substrate conversion rate

**Fig. 1:**
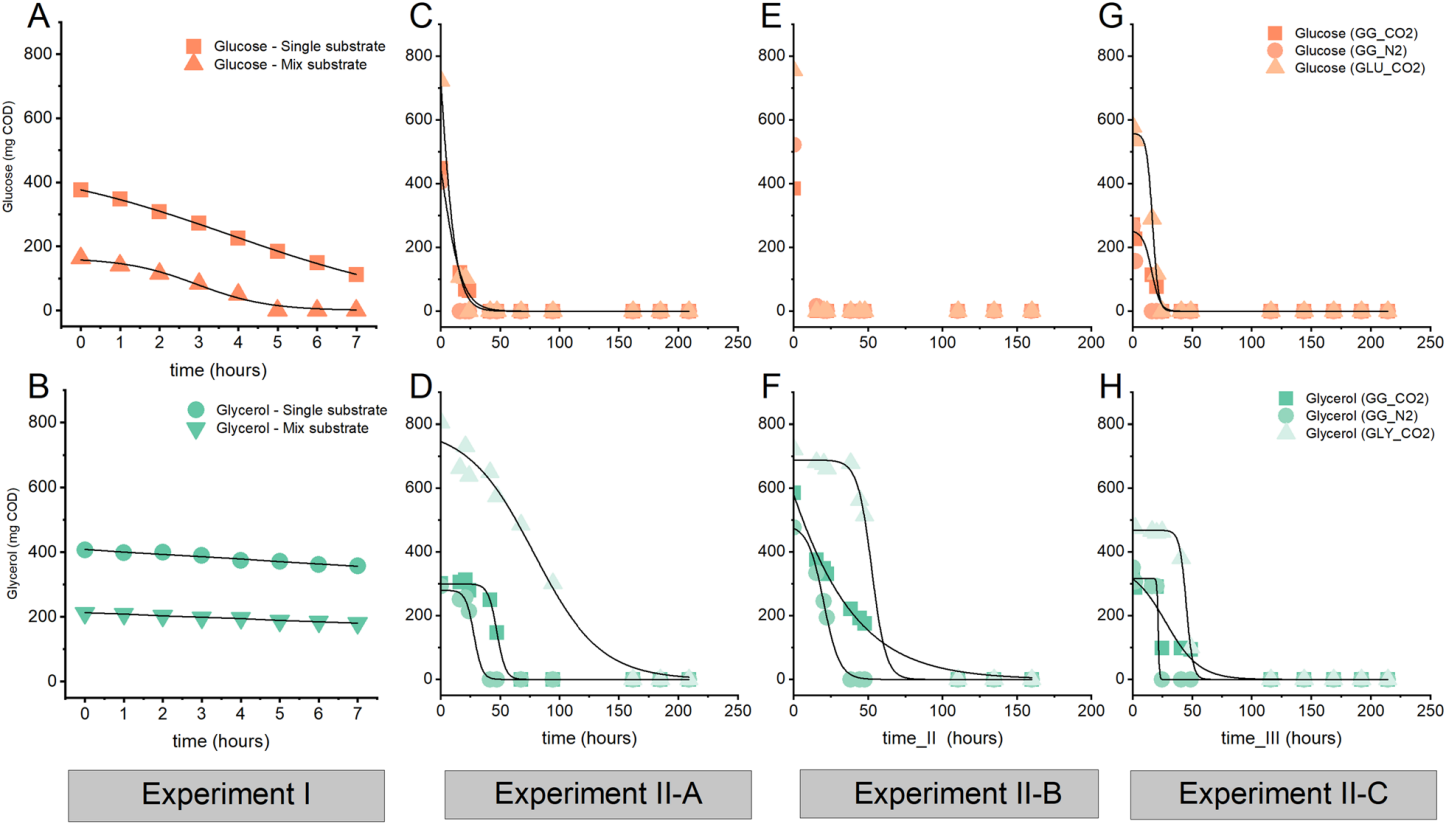
Glucose (**A**) and glycerol (**B**) conversion in single and mixed substrate treatments during Experiment I at 5 bar pCO_2_. Glucose and glycerol conversion in phases II-A (**C** and **D**), II-B (**E** and **F**) and II-C (**G** and **H**) during Experiment II. Experimental data are presented as discrete symbols for mixed substrate treatments (GG_CO2) at 5 bar pCO_2_, mix substrate control at 5 bar pN_2_ (GG_N2) and single substrate controls (glucose GLU_CO2 and glycerol GLY_CO2). Modeled data according to the description provided in Table 5 are presented as a continuous black line. Substrate is expressed in mg COD after volume correction due to liquid sampling in the reactors during each phase

In experiment I (see “Methods” section “Mixed substrate conversion under elevated pCO_2_”), the reference substrate conversion rate under single or mixed substrate at 5 bar pCO_2_ was determined using the inoculum without previous exposure to pressurized conditions. The logistic model used to describe glucose conversion showed three times faster glucose conversion at lower initial concentrations in the mixed substrate treatment than in the single substrate (Fig. 1A, Table 1). Conversely, the linear regression model showed that glycerol conversion was 1.6 times higher in the only glycerol condition than in the mixed substrate treatment (Fig. 1B, Table 1).

**Table 1:** Estimated glucose and glycerol conversion rates calculated with the logistic model (in h^−1^) and simple linear regression (in mg substrate h^−1^) for (a) treatments with single and mixed substrate in Experiment I and (b) single substrate controls at 5 bar pCO_2_ (glucose GLU_CO2 and glycerol GLY_CO2), mixed substrate control at 5 bar pN_2_ (GG_N2) and treatments with mixed substrate at 5 bar pCO_2_ (GG_CO2) in the different phases of Experiment II

Experiment	Treatment	Phase	Model	Glucose conversion rate + (adjusted *R*^2^)	Glycerol conversion rate + (adjusted *R*^2^)	Rate units
I	Single substrate—glucose		Logistic	− 0.36*** (0.95)		k (h^−1^)
	Single substrate—glycerol		Linear regression		− 7.4*** (0.96)	(mg Glycerol h^−1^)
	Mix substrate		Logistic + linear regression	− 1.10*** (0.95)	− 4.7*** (0.99)	k (h^−1^) (mg Glycerol h^−1^)
II	Single substrate—glucose (GLU_CO2)	II-A	Logistic	− 0.15** (0.99)		k (h^−1^)
		II-B		NA		
		II-C		− 0.41*** (0.99)		
	Single substrate—glycerol (GLY_CO2)	II-A			− 0.04*** (0.98)	
		II-B			− 0.21** (0.99)	
		II-C			− 0.36*** (0.99)	
	Mixed substrate (GG_CO2)	II-A		− 0.12*** (0.99)	− 0.27*** (0.99)	
		II-B		NA	− 0.03*** (0.99)	
		II-C		− 0.27** (0.98)	− 0.07* (0.89)	
	Mixed substrate (GG_N2)	II-A		NA	− 0.29** (0.99)	
		II-B		NA	− 0.19*** (0.99)	
		II-C		NA	− 1.95* (0.99)	

*NA* not available

Significance level *p < 0.05, **p < 0.01, ***p < 0.001

Substrate conversion rates were also determined in experiment II, which was carried out in sequential phases (see “Methods” section, “Mixed substrate conversion under elevated pCO_2_”): II-A (mixed substrate conversion), II-B (increase in the S/X ratio) and II-C (presence of formate as external electron donor). In phase II-A, the logistic model provided a good approximation (adjusted *R*^2^ > 0.98) to describe substrate conversion in all cases, except for glucose conversion in GG_N2. The experimental data corresponding to GG_N2 are presented in Fig. 1 (subplots C, E and G), but rates were not calculated, due to the limited number of useful data to describe the curve shape in the interval 0–50 h. Rates of glucose conversion were comparable between GG_CO2 and GLU_CO2 (Table 1). Glycerol conversion was similar between GG_CO2 and GG_N2, representing an eightfold increase compared to GLY_CO2 (Table 1). The GLY_CO2 started with a higher substrate concentration, due to a technical failure in feeding preparation, but as evidenced in Fig. 3, complete substrate depletion was achieved after 160 h. Since the standard deviation in COD fed during phase II-A was lower than 10%, reasonable comparisons between treatments and controls can be performed.

During phase II-B, glucose conversion occurred faster than in II-A, with glucose being not detectable after 20 h in all cases (Fig. 1E). Consequently, accurate rate estimation was not possible, because of the limited useful data points. Glycerol conversion rate in GG_CO2 was lower than in GG_N2 (Table 1). Conversely, rates in GLY_CO2 and GG_N2 were comparable, but differentiated by a noticeable lag phase approximately corresponding to 50 h (Fig. 1). In phase II-C, the reduction in substrate concentration and adding 5 mM formate were beneficial to achieve a faster substrate conversion. Although the glucose conversion rate in GG_CO2 was lower than GLU_CO2, both rates were higher than in phase II-A, but still with a noteworthy lag phase of approximately 20 h. Glycerol conversion rates also improved; for example, in GG_CO2, the rate was faster than in phase II-B, but remained lower than the calculated value in II-A (Table 1). In GG_N2 and GLY_CO2, the glycerol conversion rates were faster than in phases II-B and II-A (Table 1).

#### End-of-phase (EoP) product spectrum

**Fig. 2:**
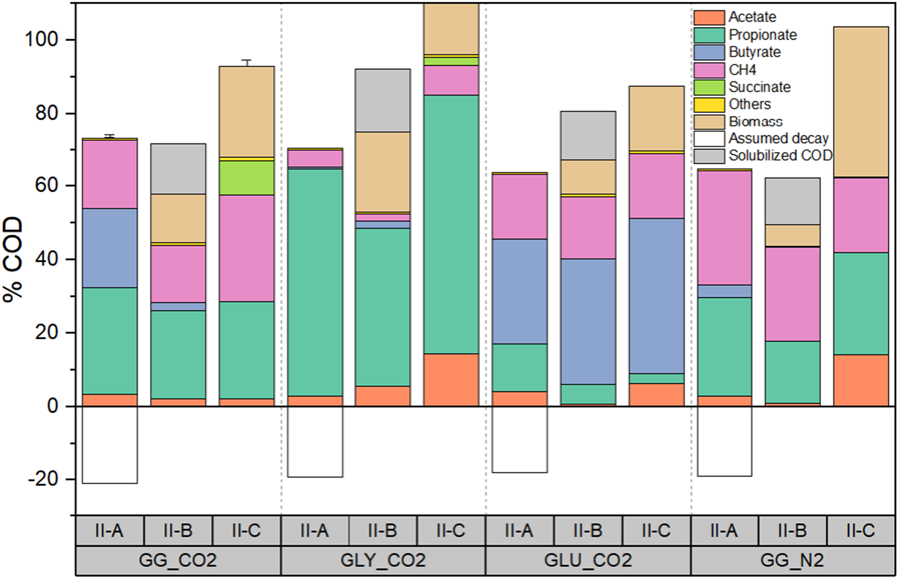
End-of-phase (EoP) product spectrum expressed as a percentage of the COD fed in phases II-A, II-B and II-C in Experiment II. Data are presented for the mixed substrate treatments (GG_CO2) at 5 bar pCO_2_, mixed substrate control at 5 bar pN_2_ (GG_N2) and single substrate controls (glucose GLU_CO2 and glycerol GLY_CO2) at 5 bar pCO_2_

**Fig. 3:**
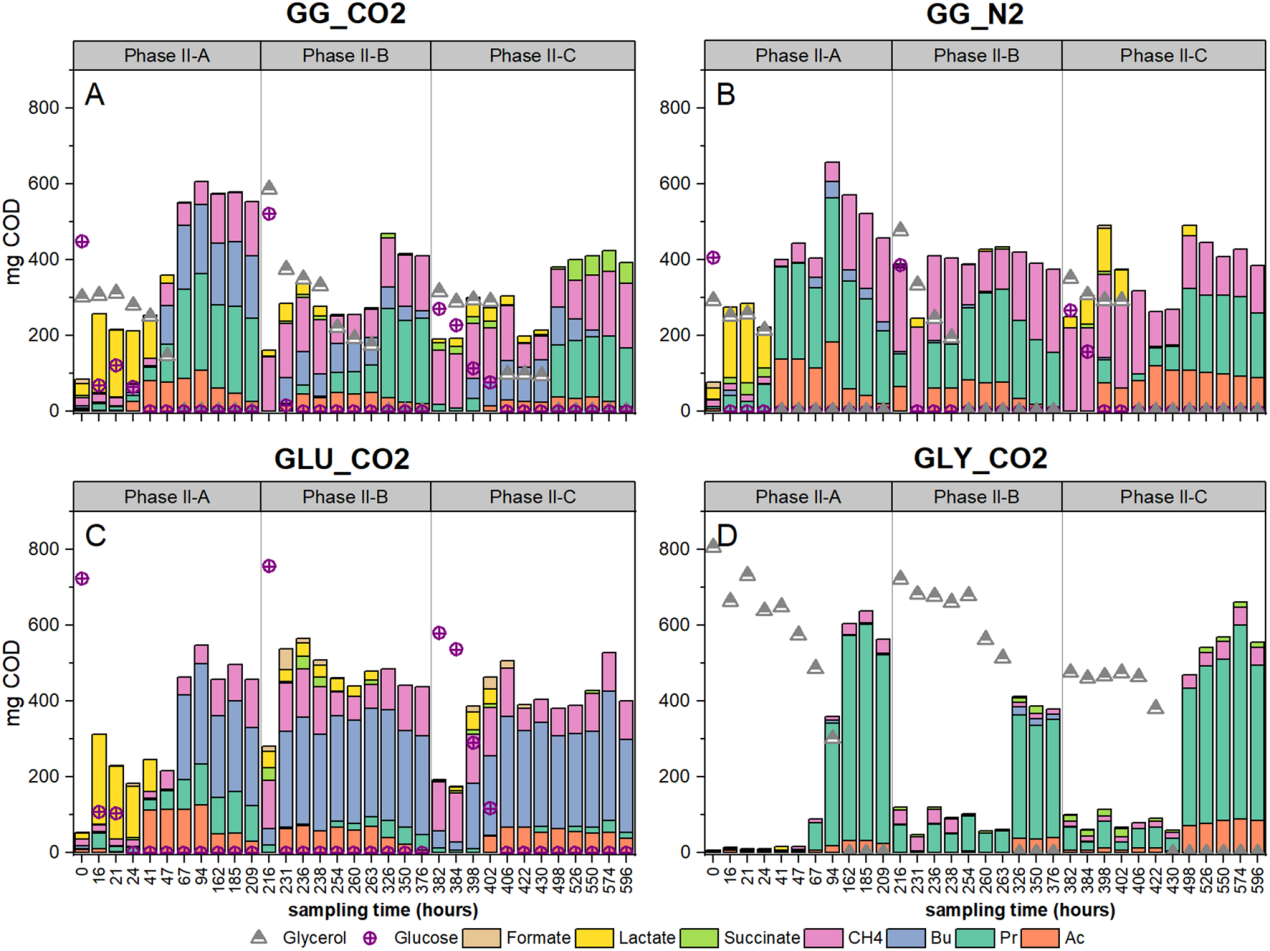
Product spectrum (values in the *y*-axis expressed in mg COD) over time in the different phases (II-A, II-B and II-C) of Experiment II. Experiment II lasted 596 h and values in the *x*-axis correspond to the different sampling points. Data are presented for mixed substrate treatments at 5 bar pCO_2_ (GG_CO2) (**A**), mixed substrate control at 5 bar pN_2_ (GG_N2) (**B**) and single substrate controls (glucose GLU_CO2 and glycerol GLY_CO2) (**C** and **D**). Abbreviations correspond to methane (CH_4_), butyrate (Bu), propionate (Pr) and acetate (Ac)

In phase II-A, COD was converted to lactate (28–33%) during the first 21 h in all treatments and controls, except for GLY_CO2 (< 1%). Intermediate succinate and formate represented less than 2% of the COD fed in all cases, except for GG_N2 (5%) (Fig. 3B). After 209 h, the EoP product spectrum for GG_CO2 was composed of propionate (30 ± 5%), butyrate (22 ± 4%) and acetate (3 ± 1%) (Fig. 2, expressed in mg COD). The EoP product spectrum in GG_N2 showed similarities with GG_CO2, except for a low butyrate contribution (3%) (Fig. 2). In the case of GLY_CO2, the EoP product spectrum was dominated by propionate (62%), with a small acetate proportion (3%) (Fig. 2); whereas GLU_CO2 showed a high proportion of butyrate (29%) and a lower proportion of propionate (13%) and acetate (4%) (Fig. 2). Biomass decay, calculated as the difference between initial and final VSS concentrations, occurred in all treatments during phase II-A (Fig. 2). Regarding the EoP gaseous products, in GG_CO2, approximately 19 ± 3% of the COD-fed accounted as CH_4_. In GG_N2, COD-CH_4_ corresponded to 32% of COD-fed, and for GLY_CO2 and GLU_CO2 accounted for 5 and 18%, respectively.

Increased substrate concentrations during phase II-B did not cause important changes in the EoP product spectrum in GG_CO2. After correcting for carried-over concentration from phase II-A, EoP product spectrum was composed of propionate (24 ± 2%), butyrate (2 ± 0%) and acetate (2 ± 0%) (Fig. 2). In GG_N2, it was composed of propionate (17%) and acetate (1%), while butyrate was not detected (Fig. 2). In GLY_CO2, COD was primarily transformed to propionate (43%), whereas in GLU_CO2, to a mixture of butyrate (35%), propionate (6%) and acetate (1%) (Fig. 2). The CH_4_ production accounted for 15 ± 2%, 2 and 17% of the COD-fed in the case of GG_CO2, GLY_CO2 and GLU_CO2, respectively. The control GG_N2 showed a decrease in the recovery of COD as CH_4_ (only 15%).

Noteworthy differences were observed in the EoP product spectrum after adding formate and reducing the substrate concentration to reduce the S/X ratio (phase II-C). An increase in succinate production (10 ± 2% from COD fed) was detected in GG_CO2 (Fig. 2). Succinate accumulated until the end of phase II-C after 214 h. Lactate (8 ± 2%) was detected in the first 16 h and was further converted to carboxylates (Fig. 3). The EoP product spectrum in GG_CO2 included propionate (27 ± 7%) and acetate (2 ± 0.4%), while no butyrate was detected (Fig. 2). The control GG_N2 did not show the same trend regarding lactate and succinate. Initially, 18% of the COD was directed to lactate and only 2% to succinate (Fig. 3). By the end of phase II-C, both metabolites were not detected. The EoP product spectrum in GG_N2 was composed of propionate (28%), acetate (14%) but no additional butyrate. GLY_CO2 showed a COD recovery of 112% after discounting for carried-over COD from phase II-B (Table 2) and its EoP product spectrum included propionate (85%) and acetate (17%). In the control GLU_CO2, the EoP product spectrum corresponded to butyrate (42%), propionate (3%) and acetate (6%). An increase in acetate was observed in all conditions except for GG_CO2, which showed acetate levels comparable to phases II-A and II-B (Fig. 2). CH_4_ production in GG_CO2 accounted for 29 ± 10% of the COD-fed, whereas in GLY_CO2, GLU_CO2 and GG_N2, it accounted for 10, 18 and 20% of the COD fed, respectively.

**Table 2:** The COD balance for phases II-A, II-B and II-C in Experiment II*

Sample	Phase	Acetate (mg COD)	Propionate (mg COD)	Butyrate (mg COD)	CH_4_ (mg COD)	Succinate (mg COD)	Others (mg COD)	Biomass^a^ (mg COD)	Total (mg COD)	COD fed (mg)	Recovered fraction from COD substrate^b^	Recovered fraction from COD substrate (included solubilized COD)^c^	COD fed + carry over from previous phase (mg)	Recovered fraction from COD substrate + carry over^d^
GG_CO2 (*n* = 3)	II-A	25.26 ± 8.6	221.18 ± 31.3	163.86 ± 31.3	142.80 ± 23.9	0.00 ± 0.0	3.31 ± 3.1	0.00 ± 0.0	556.41 ± 56.7	762 ± 28	0.73 ± 0.1	0.73 ± 0.1	779.21 ± 26.9	0.71 ± 0.1
	II-B	20.57 ± 2.9	224.44 ± 32.9	20.11 ± 59.9	144.51 ± 16.2	0.00 ± 0.0	7.06 ± 2.3	123.92 ± 45.2	540.60 ± 88.6	935 ± 143	0.58 ± 0.1	0.71 ± 0.1	1150.08 ± 78.6	0.47 ± 0.1
	II-C	12.26 ± 2.4	155.70 ± 41.5	0.00 ± 0.0	169.66 ± 70.7	55.20 ± 6.4	5.50 ± 2.7	147.11 ± 42.0	545.43 ± 142.9	586 ± 40	0.93 ± 0.3	0.93 ± 0.3	1032.26 ± 65.3	0.53 ± 0.1
GLY_CO2 (*n* = 1)	II-A	24.39	497.24	4.53	36.86	0.00	3.36	0.00	566.38	806	0.70	0.70	814.03	0.70
	II-B	40.18	310.46	14.02	13.88	0.00	3.44	158.36	540.34	722	0.75	0.92	1226.81	0.44
	II-C	84.02	410.60	0.00	47.28	13.53	2.92	94.42	652.79	582	1.12	1.12	1129.64	0.53
GLU_CO2 (*n* = 1)	II-A	29.89	94.19	206.14	126.83	0.00	4.83	0.00	461.89	724	0.64	0.64	745.14	0.62
	II-B	6.44	41.18	261.65	128.86	0.00	5.97	71.45	515.56	768	0.67	0.81	1098.94	0.47
	II-C	36.91	16.00	244.83	101.94	0.00	4.79	103.14	507.60	581	0.87	0.87	1071.78	0.47
GG_N2 (*n* = 1)	II-A	20.38	191.72	23.73	222.17	0.00	3.34	0.00	461.34	713	0.65	0.65	728.69	0.63
	II-B	9.14	145.75	0.00	219.17	0.00	2.57	50.69	427.32	862	0.50	0.62	1216.32	0.35
	II-C	87.79	171.39	0.00	124.81	0.00	2.57	253.33	639.89	617	1.04	1.04	846.69	0.76

*Data are presented for mix substrate treatments (GG_CO2) at 5 bar pCO_2_, mix substrate control at 5 bar pN_2_ (GG_N2) and single substrate controls (glucose GLU_CO2 and glycerol GLY_CO2). The standard deviation of three biological replicates is included only in the case of GG_CO2 treatments

^a^Biomass was calculated as the difference between initial and final VSS concentration in each phase and converted to COD using a conversion factor of 1.42 g COD/g VSS

^b^This fraction corresponds to the COD recovered as end of phase (EoP) products considering only the soluble COD from the substrate

^c^This fraction corresponds to the COD recovered as end of phase (EoP) products considering that a theoretical 80% of the COD from decay in phase II-A became solubilized into phase II-B

^d^This fraction corresponds to the COD recovered as end of phase (EoP) products considering COD from substrate and carried-over carboxylates/organic acids from the immediate previous phase

In GG_CO2 treatments, high propionate production was hypothesized as an effect of mixed substrate and elevated pCO_2_ during experiment II; however, there were no significant differences (*p* = 0.32) in propionate concentrations between GG_CO2 and GG_N2 (paired samples Wilcoxson test). In contrast, butyrate concentrations were significantly different (*p* < 0.0001) in all phases of experiment II. The aligned rank transform test was used to evaluate if independent factors, e.g., process conditions or their interaction effects were significant to explain the differences in carboxylate concentrations and cell density in experiment II. When analyzing pCO_2_ as the main effect, this factor was non-significant for carboxylates and succinate production, as evidenced by the *p*-values (Table 3). In contrast, the interaction effect between pCO_2_ and process conditions was significant to explain differences in propionate and butyrate production (pCO_2_–substrate concentration) and succinate production (pCO_2_–additional electron donor) (Table 3).

**Table 3:** Summary of p-values^a^ from the aligned rank transform test for the main and interaction effects of elevated partial pressure of CO_2_ (pCO_2_) or N_2_ (pN_2_), substrate concentration and addition of external electron donor (formate) in carboxylates production and cell density during Experiment II

Independent factor		pCO_*2*_		Substrate concentration (S/X ratio)	pCO_2_		External electron donor
Interaction			pCO_*2*_: substrate concentration (S/X ratio)			pCO2: external electron donor	
Dependent variable	Propionate (mg COD L^−1^)	0.81	0.94	****	0.78	0.07	0.59
	Butyrate (mg COD L^−1^)	0.37	****	****	0.62	0.11	0.76
	Acetate (mg COD L^−1^)	0.25	0.31	0.13	0.31	0.43	0.36
	Succinate (mg COD L^−1^)	0.22	0.18	0.67	0.26	****	****
	Cell density (cells m L^−1^)	0.77	0.09	*	0.87	0.75	****

^a^Significance level *p < 0.05, **p < 0.01, ***p < 0.001, ****p < 0.0001

#### Cell density

**Fig. 4:**
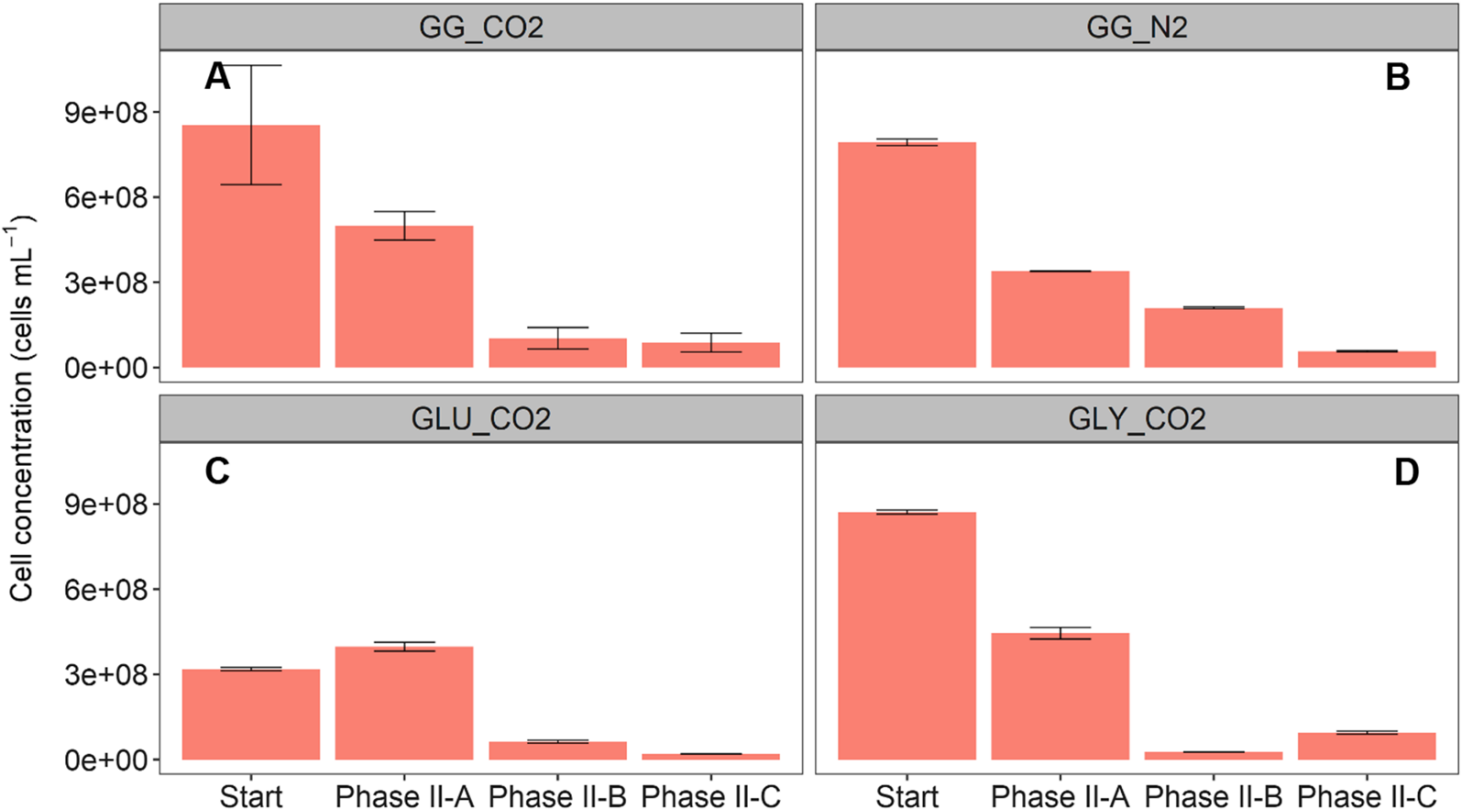
Total cell density measured at the beginning and the end of the different phases of experiment II. Data correspond to treatments with mixed substrate (glycerol–glucose GG_CO2) at 5 bar pCO_2_, control at 5 bar pN_2_ (GG_N2) and controls for conversion of individual substrate as glucose (GLU_CO2) and glycerol (GLY_CO2) at 5 bar pCO_2_. Error bars represent the standard deviation of three technical replicates employed during flow cytometry (FCM)

During phase II-A, a decrease in total cell density of 42 ± 14, 49 and 57% was established for GG_CO2, GLY_CO2 and GG_N2, respectively, whereas GLU_CO2 showed a moderate increase (25%) (Fig. 4). In phase II-B, the measured cell density was corrected for dilution, due to medium refreshing. A steep decrease of 70 ± 8% in cell density was observed in GG_CO2, compared to initial values. A similar sharp decrease was registered in reactors GLY_CO2 and GLU_CO2, corresponding to 91 and 76%, respectively. Conversely, GG_N2 showed a slight reduction (7%).

The cell density was positively affected by formate addition and reduction in substrate concentration to lower the S/X ratio in phase II-C, with the final biomass in GG_CO2 increasing by 28 ± 5%. In GLY_CO2, there was an even more predominant positive effect, since cell density experienced a fivefold increase compared to the initial concentration (Fig. 4D). However, this effect did not appear in GLU_CO2, where a substantial decrease in cell density was assessed, i.e., 53%. A similar observation was registered for control GG_N2, where the cell density decreased by 59%, higher than values observed in phase II-B.

Cell densities were significantly different between the start and end points of phase II-A (*p* = 0.0008), II-B (*p* = 0.0003) and II-C (*p* = 0.00001). The aligned rank transform test showed that substrate concentration and addition of external electron donor were the main effects explaining the differences in cell density, whereas pCO_2_ did not. However, the interaction effects pCO_2_–substrate concentration and pCO_2_–additional electron donor were significant to explain differences in cell densities in experiment II (Table 3).

### Changes in the microbial community

During experiment II, the effect of sequential changes in operational conditions on microbial community structure and its relation with shifts in product spectrum under elevated pCO_2_ was examined. Microbial community analysis resulted in an average of 140,365 ± 6153 total reads per sample and 2230 OTUs in total. After singleton removal, OTUs were reduced to 1935. Bacteria and Archaea corresponded to 80% and 20% of total processed reads in the original inoculum. The bacterial community in the inoculum was composed of Anaerolineae (77%), Actinobacteria (6%) and Clostridia (5%) at the class level. The archaeal community in the inoculum primarily included Methanomicrobia (81%) and Methanobacteria (19%). Changes in the relative abundance of Bacteria and Archaea during experiment II are presented in Additional file 1: Table S1.

**Fig. 5:**
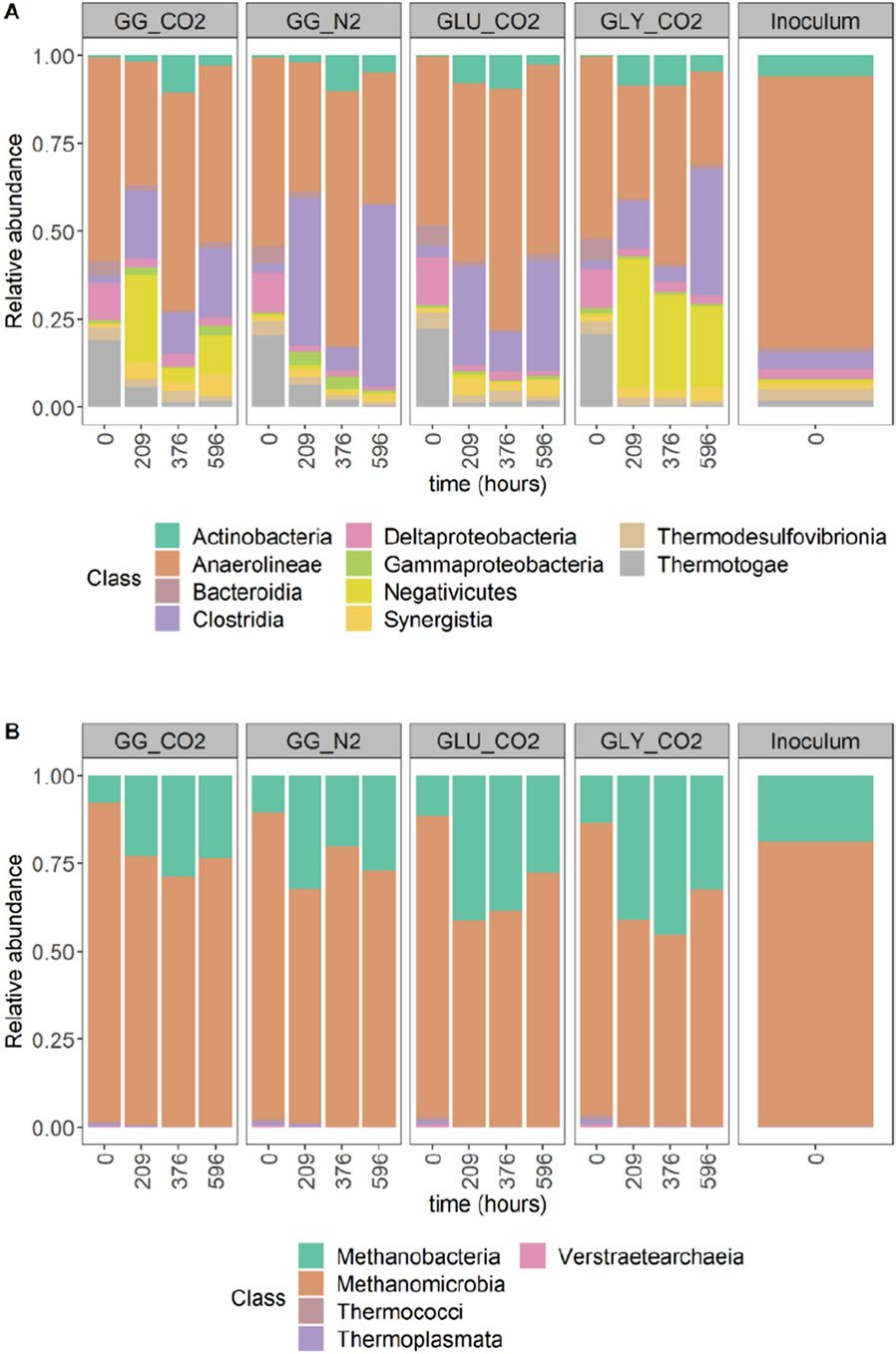
Relative abundances of the top 10 most abundant classes across all treatment and control samples in **A** bacterial community and the top 5 most abundant classes in **B** archaeal community. The horizontal axis includes the time points where samples were taken and correspond to the start of experiment II and the end of phase II-A (209 h), II-B (376 h) and II-C (596 h)

In phase II-A, the bacterial community of GG_CO2 was mainly composed of the class Anaerolineae (35 ± 8%), Negativicutes (family Veillonellaceae) (27 ± 20%), Clostridia (19 ± 16%) and Thermotogae (6 ± 0%). The GG_N2 included Clostridia (42%), Anaerolineae (37%) and Thermotogae (7%). GLY_CO2 comprised Negativicutes (37%), Anaerolineae (32%) and Clostridia (13%). GLU_CO2 showed a predominance of Anaerolineae (51%), Clostridia (28%) and Actinobacteria (8%) (Fig. 5A). In phase II-B, Anaerolineae became predominant in GG_CO2 (62 ± 1%), being followed by Actinobacteria (11 ± 1%) and Clostridia (12 ± 2%). GG_N2 showed an almost complete predominance of Anaerolineae (72%). The major classes in GLY_CO2 corresponded to Anaerolineae (51%), Negativicutes (27%) and in GLU_CO2 to Anaerolineae (69%), Clostridia (11%) and Actinobacteria (9%) (Fig. 5A). Noticeable changes in the community were observed in phase II-C: GG_CO2 still showed Anaerolineae predominance (50 ± 6%), but the abundance of Clostridia (20 ± 14%) and Negativicutes (11 ± 9%) increased. Synergistia (6 ± 4%) also showed higher abundance than phases II-A and II-B. In GG_N2, Clostridia (52%) and Anaerolineae (37%) were predominant and Synergistia exhibited low abundance (2%). Clostridia (36%) dominated in GLY_CO2, followed closely by Anaerolineae (27%), Negativicutes (23%) and Synergistia corresponded to 4%. In GLU_CO2, bacterial community composition resembled phase II-A: Anaerolinea (54%), Clostridia (32%), but Synergistia accounted for 5% (Fig. 5A).

The dominant classes in the archaeal community were Methanomicrobia and Methanobacteria, with fluctuating abundances throughout experiment II. After phase II-A, Methanomicrobia and Methanobacteria accounted for 76 ± 4% and 23 ± 4% in GG_CO2. In GG_N2, their abundances were 66 and 32%, respectively. In GLY_CO2 and GLU_CO2, abundances were similar: 59% vs. 41% (Fig. 5B). In phase II-B, abundances remained comparable to phase II-A for GG_CO2, GLY_CO2 and GLU_CO2. However, GG_N2 showed an increased abundance of Methanomicrobia (80%) (Fig. 5B). In phase II-C, some slight changes in the abundance were observed. Methanomicrobia corresponded to 76 ± 1% and Methanobacteria to 23 ± 1% in GG_CO2. Relative abundances of both families in GG_N2, GLU_CO2 and GLY_CO2, corresponded to 73–27%, 67–32% and 72–28%, respectively (Fig. 5B). The trends in group predominance in the bacterial and archaeal communities were also validated by absolute taxon abundance with flow cytometry data according to [54] to highlight the effect of inter-sample changes in cell density during experiment II (Additional file 1: Fig. S1.

**Fig. 6:**
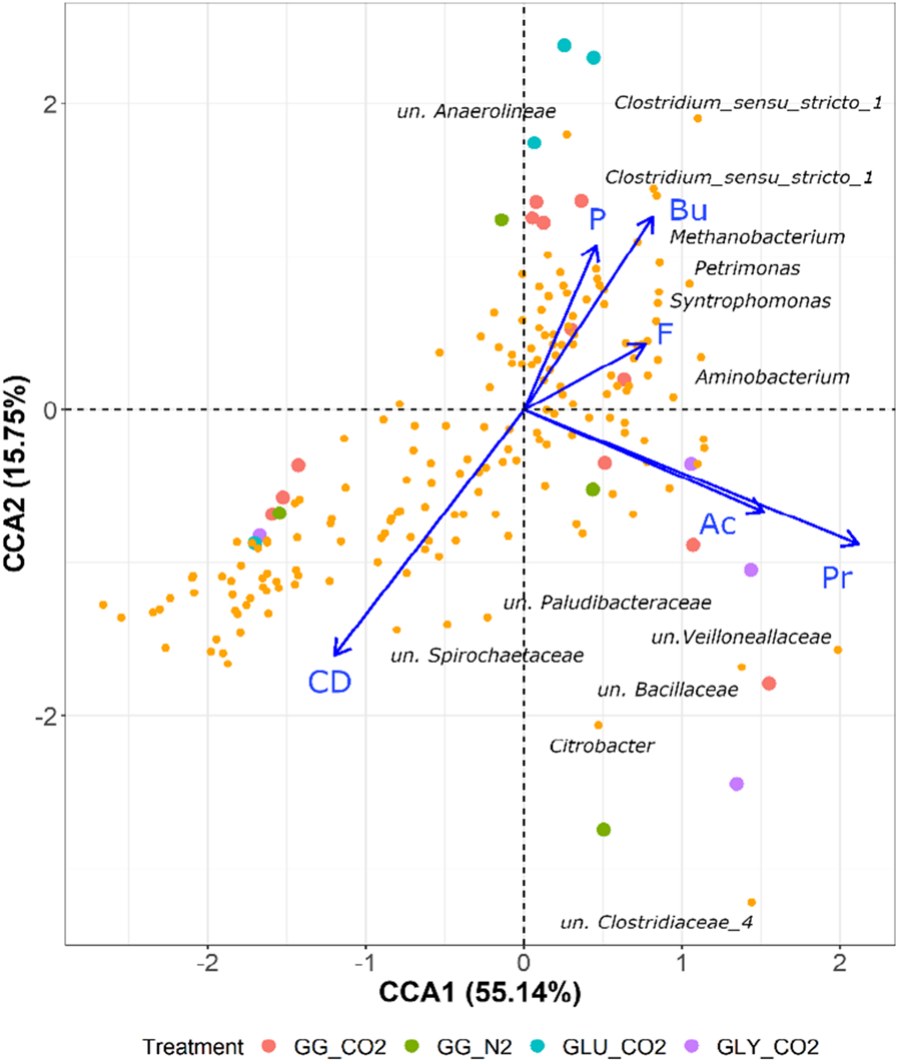
Canonical correspondence analysis (CCA) of the microbial community ordinated at the OTU level. Samples correspond to mixed substrate (GG_CO2) at 5 bar pCO_2_, mixed substrate control at 5 bar pN_2_ (GG_N2) and single substrate controls (glucose GLU_CO2 and glycerol GLY_CO2). Significant correlations between community composition and operational conditions: elevated pCO_2_ (P), presence of formate as additional electron donor (F), carboxylate concentrations (acetate (Ac), propionate (Pr), butyrate (Bu)) and cell density concentrations (CD normalized to the log_10_) are depicted as blue arrows

Significant differences in richness were only found in the archaeal community (*p* = 0.005) when comparing the initial and final stages in experiment II, and not in the bacterial community (*p* = 0.18). In terms of the beta diversity, significant differences were found between the archaeal community in the inoculum and the sample used as starting point for experiment II, indicated as *t* = 0 in Fig. 5 (*p* = 0.03), which suggests an effect of sample storage. However, this was not the case for the bacterial communities (*p* = 0.14). On the other hand, significant differences were not found between the bacterial and archaeal community when grouped by type of substrate (*p* = 0.78 and *p* = 0.83) and gas used for headspace pressurization (*p* = 0.63 and *p* = 0.85), respectively. However, bacterial and archaeal communities were significantly different when considering the presence and absence of formate as an additional electron donor (*p* = 0.001 and *p* = 0.03). Total cell density, as the *proxy* of biomass growth, measured carboxylate concentrations, external electron donor (formate) and pCO_2_ were included in a canonical correspondence analysis (CCA) to highlight possible correlations between process conditions and microbial community structure. The constrained variables selected for constructing the model explained 70% (*p* = 0.001) of the variance in the microbial community (Fig. 6).

### Undissociated carboxylic acids

**Fig. 7:**
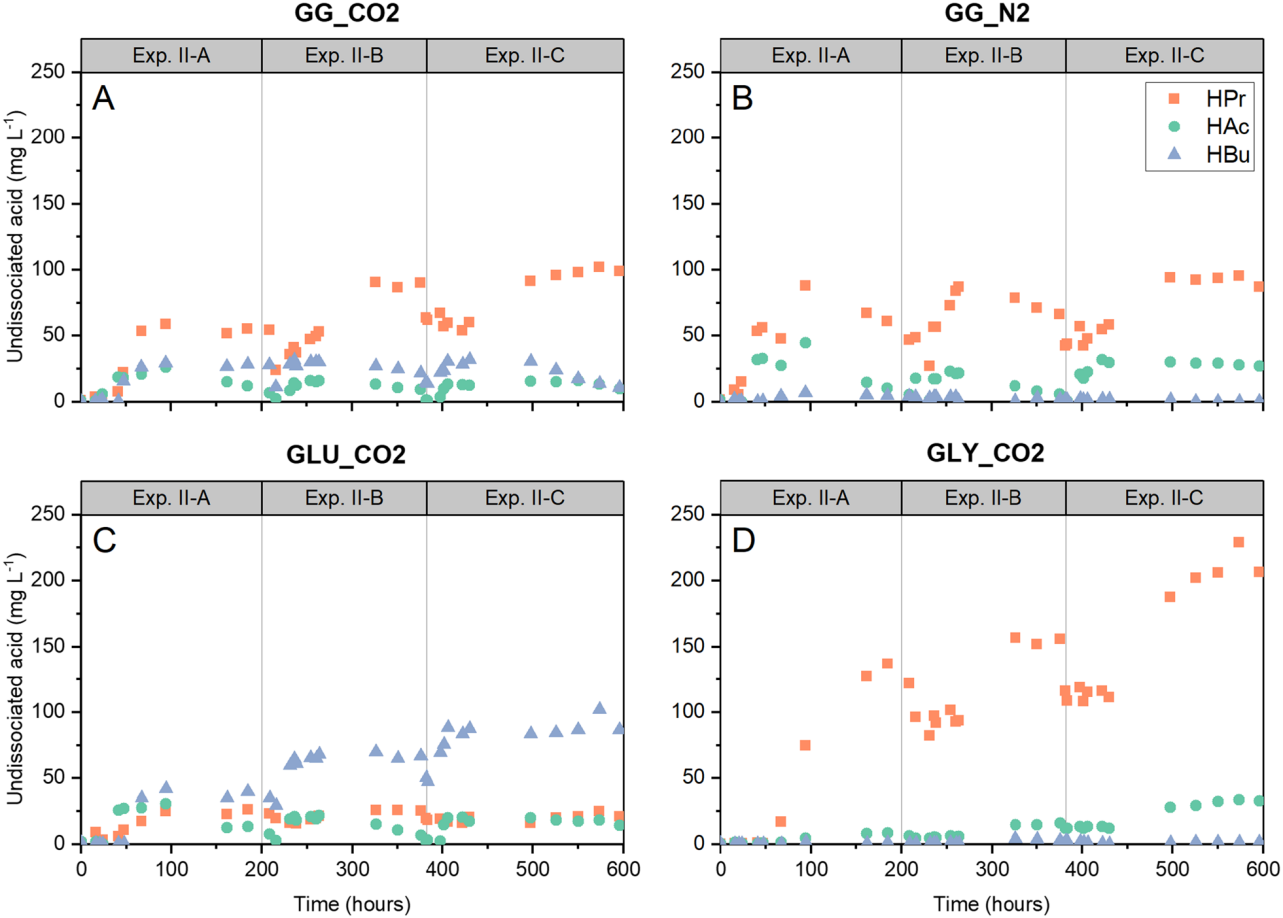
Evolution of calculated undissociated carboxylic acid concentrations (acetic—HAc, propionic—HPr and butyric—HBu acids) during experiment II. Data are presented for mixed substrate treatments at 5 bar pCO2 (GG_CO2) (**A**), mixed substrate control at 5 bar pN2 (GG_N2) (**B**) and single substrate controls (glucose GLU_CO2 and glycerol GLY_CO2) (**C** and **D**)

High substrate concentration leading to an increase in the S/X ratio (Phase II-B) could be compatible with enhanced acid production, due to disparities in acid production and consumption. Combined with CO_2_ dissolution in each phase, it could have led to pH fluctuations, despite the provided high buffer concentration. Lowered pH could provoke an increase in the concentration of undissociated carboxylic acids, which could inhibit microbial activity. A pH value of 6.5 was calculated (“Methods” Eqs. 2 and 3) as the lowest equilibrium value achieved in phase II-B, and we used it to estimate the time evolution of undissociated acetic (HAc), propionic (HPr) and butyric (HBu) acids throughout experiment II as depicted in Fig. 7. Additionally, undissociated carboxylic acid concentrations were employed to investigate possible correlations with changes in the microbial community structure. A significant negative correlation between total undissociated carboxylic acids (expressed as mg HAc equivalents L^−1^) and the log-absolute abundance of total archaea (*r*_s_ = − 0.71, *p* = 0.002) was found (Additional file 1: Fig. S2A). Both predominant classes, Methanomicrobia and Methanobacteria, were negatively correlated with undissociated carboxylic acids (*r*_s_ = − 0.73, *p* = 0.001 and *r*_s_ = − 0.58, *p* = 0.019) (Additional file 1: Fig. S2B, C).

## Discussion

### Interaction effects between elevated pCO_2_–operational conditions steer product formation in anaerobic mixed culture fermentation

Previous research has suggested a potential steering role of elevated pCO_2_ in anaerobic processes, e.g., AD and mixed culture fermentation, particularly concerning propionate production [26]. Observed differences in the production/conversion of propionate and butyrate in GG_CO2 and GG_N2 during experiment II align with this proposition. However, these differences cannot be exclusively ascribed to elevated pCO_2_, since there were also disparities in methanogenic activity between GG_CO2 and GG_N2 (Table 1), which could have led to the accumulation of reducing equivalents, eventually impacting pathway feasibility. Higher CH_4_ production occurred in GG_N2 than in GG_CO2 during phases II-A and II-B (Fig. 2A, B), which aligns with results previously presented for glucose and glycerol conversion with non-adapted inoculum at 5 bar pN_2_/pCO_2_ [55]. Our previous work [51] reported a reduction (53 to 85%) in the methane yields when reactor headspace was pressurized with CO_2_ rather than N_2_. However, in phase II-C, the obtained CH_4_ became similar in GG_CO2 and GG_N2 due to a decrease in the methanogenic activity in GG_N2 (Table 3) and resumed methanogenic activity in GG_CO2 after decreasing the S/X ratio and adding formate (Fig. 2A, B). These changes in methanogenic activity occurred despite the overall reduction in absolute archaeal abundances (Additional file 1: Fig. S1B).

Several roles can be attributed to formate in the methanogenesis reestablishment during phase II-C. Formate can be indistinctively used by some hydrogenotrophic methanogens [56, 57] and acetogenic bacteria, as an electron donor [58]. Acetogenic bacteria have shown a higher formate affinity than methanogens when cultivated together; thus, at low formate concentrations, acetogenic bacteria could outcompete methanogens regarding formate utilization [59]. Under conditions of elevated pCO_2_, methanogenesis in GG_CO2 could have been re-established via a “mediated” process. Acetogenic bacteria could have utilized formate to fix CO_2_ into acetate, which, together with acetate from glucose fermentation (Eqs. 3, 4, 6 Additional file 1: Table S2), was subsequently consumed by acetotrophic methanogens. The concomitant increase in the absolute abundance of *Clostridium* and *Veillonella* spp. and class Methanomicrobia (genus *Methanosaeta*) in treatments GG_CO2 during phase II-C supports this postulate (Fig. 5). It has been reported that low formate concentrations (2 mM) can have notorious effects on microbial growth and activity [58, 60]; but also microbial acclimation after short-term sequential batch incubation [61] could have played a role in the observed differences between phase II-A and II-C in the treatments of experiment II. More experimental work including incubations in parallel is required to differentiate effects associated with formate addition and microbial acclimation.

The product spectrum in GG_CO2 closely resembled the expected profile from the independent conversion of glycerol and glucose at 5 bar pCO_2_, with propionate and butyrate being present (Fig. 2). Butyrate rather than propionate concentrations were significantly different between GG_CO2 and GG_N2 (see Results). At first instance, this result diverges from observations presented earlier [26, 55], and it does not align with a direct link between elevated pCO_2_ and, e.g., high propionate levels. However, this finding evidences that the relation pCO_2_–product spectrum is more complex than initially proposed. The product profile does not seem to be defined by an isolated effect of pCO_2_, but rather by interaction effects with operational conditions, such as type of substrate, S/X ratio and presence of additional electron donors (Table 3). Furthermore, these changes in the intermediate product spectrum result from a trade-off in terms of reduced substrate consumption rate and establishment of lag-phases due to elevated pCO_2_ (Fig. 1, Table 1).

When analyzing the product spectrum for the individual substrates, enhanced propionate production from glycerol was observed in control GLY_CO2 at 5 bar pCO_2_ (Fig. 2). Elevated pCO_2_ most likely stimulated carbon fixation in the reductive side of the TCA cycle towards propionate via the higher activity of enzymes, such as pyruvate carboxylase [62], CoA transferase [63] and PEP carboxykinase [64]. In contrast to results presented earlier [26, 55], where propionate was the main metabolite from glucose conversion under 5.0 and 6.2 bar pCO_2_, respectively, GLU_CO2 presented high butyrate and acetate levels. Increased butyrate production in GLU_CO2 and GG_CO2 could be attributed to the presence of excess acetate and carbohydrates enhancing the activity of butyryl-CoA:acetate CoA-transferase, a key enzyme in the pathway of certain *Clostridium* spp. [65] (Eq. 5 Additional file 1: Table S2). *Clostridium* spp. were a predominant bacterial group in GLU_CO2 (Fig. 5A) and showed a strong positive correlation with butyrate concentrations (Fig. 6). However, they were less predominant in prior glucose experiments [55] and DGGE results from [26], which helps to explain differences in the reported product spectrum composition. Excess acetate may be ascribed to reduced consumption by acetotrophic methanogens, since absolute archaeal abundances were negatively impacted by elevated CO_2_ (Additional file 1: Fig. S1B), high S/X ratio (phase II-B) and increasing concentrations of undissociated carboxylic acids (Fig. 7).

In GG_CO2_,_ succinate production may have become a suitable alternative to dispose of excess reducing equivalents after formate addition if propionate conversion became limited by the predicted high concentrations of undissociated carboxylic acids [66] (Fig. 7). Stimulating carboxylation activity in pyruvate carboxylase and PEP carboxykinase by elevated CO_2_ concentrations has shown positive correlations with succinate production [67]. In pure cultures of *Actinobacillus succinogenes*, keeping CO_2_ concentrations above 17.1% saturation increased succinate levels [68]. Besides CO_2_ supply, providing an additional electron donor (in this case, formate) has been favorable for succinate production [37]. As an intermediate metabolite in pressurized anaerobic systems, succinate has only been previously reported by [69] as a product of the saccharification of gelatinized starch at 30 °C and 16 bar. Thus, despite yields being low, i.e., succinate represented < 10% of the COD fed to GG_CO2 in phase II_C (Table 1), results constitute encouraging evidence of the steering potential of elevated pCO_2_.

Other changes in product spectrum after formate addition in phase II-C were related to higher acetate concentrations in the controls (Fig. 2). Our results suggest, through indirect observations, the occurrence of enhanced CO_2_ fixation in phase II-C, due to formate addition. First, a considerable increase in COD-acetate in GLU_CO2 and particularly in GLY_CO2 was observed (Table 2). Second, significant differences were detected in the absolute abundances of bacteria with possible acetogenic metabolism in the Firmicutes phylum, such as *Clostridium* spp., (*p* < 0.05). Third, an increased relative abundance of *Methanosaeta* (Fig. 5B) in response to higher acetate levels with concomitant recovered CH_4_ production in all pCO_2_ treatments (Table 2). Finally, high cell densities in GG_CO2 and GLY_CO2 could have resulted from carbon and electron fluxes being directed towards anabolic processes in response to enhanced CO_2_ fixation in the presence of organic non-methanized substrate (formate) as electron donor (Fig. 4A and D). However, definite proof of enhanced CO_2_ fixation shall come from studies with labeled formate and CO_2_ to track the carbon faith on pressurized anaerobic conversions. In this way, it will be possible to differentiate between acetate from CO_2_ fixation and acetate accumulated because of reduced methanogenic activity. Moreover, substrate-labeled studies can be complemented with genome-centric metatranscriptomics analysis to examine the up-regulation of, e.g., genes of the Wood–Ljungdahl pathway in metagenome-assembled genomes [70].

Other differences between GG_CO2 and GG_N2 corresponded to measured cell densities and biomass-related products. After two pressurization cycles and formate addition in phase II-C, final cell density (*proxy* of biomass growth) was 1.5 and 4.5 times higher in GG_CO2 than in controls GG_N2 and GLU_CO2, respectively (Fig. 4). According to the aligned rank transform test, the presence of an additional electron donor was a significant factor to explain differences in cell density (Table 3). The additional availability of reducing equivalents from formate could have enhanced CO_2_ fixation and, in turn, increased levels of acetyl-CoA, a precursor of anabolic and catabolic products [71]. Remarkably, there was an opposite trend between the results from cell density measurements and VSS concentrations in experiment II. When analyzing VSS (expressed as COD-biomass), the values for GG_N2 were the ones 1.7, 2.7 and 2.5 times higher than GG_CO2, GLY_CO2 and GLU_CO2 in phase II-C, respectively (Table 2). These results suggest that VSS may not constitute an adequate *proxy* of biomass growth, since it does not distinguish between dead/non-viable cells, extracellular compounds and the biomass corresponding to active microbial cells [36, 72]. Further research shall systematically quantify biomass and biomass-associated products under pressurized headspace to discriminate between enhanced anabolism and higher synthesis of extracellular microbial products.

### Interaction effects between elevated pCO_2_–process conditions modify the community composition and indirectly product spectrum

The interaction effects between pCO_2_–operational conditions were expected to modify the metabolic activity and microbial community structure, indirectly impacting the product spectrum. Particularly, under unfavorable conditions for methanogenesis, such as high S/X ratio, increased undissociated carboxylic acids and elevated pCO_2_, an increase in the abundance of stress-tolerant microorganisms with metabolic flexibility was expected [73]. The absolute abundances of the par excellence stress-tolerant *Clostridium* spp. (Additional file 1: Fig. S1) were significantly different (*p* = 0.001) when considering phase II-A, II-B and II-C as grouping factor in experiment II. Previous investigations have shown positive effects of formate addition on the growth rates and total carbon fixation of certain acetogenic cultures, particularly in *Clostridium* spp. such as *C. ljungdahlii* and *C. carboxidivorans* [58, 74], which aligns with the changes of *Clostridium* spp. in phase II-C. Metabolic pathways for carbohydrate fermentation in *Clostridium* spp. are diverse. At low pH, ABE fermentation, i.e., acetone–butanol–ethanol [75] occurs, whereas, at circumneutral pH, the fermentation pattern could be dominated by butyrate-acetate [76]. Propionate production in *Clostridium* spp. is ascribed to the acrylate pathway, i.e., lactate to propionate [77], occurs in particular strains (*C. propionicum)* and has been evidenced using glucose or glycerol as substrates [78]. Hence, provided that *Clostridium* spp. with propionate-producing metabolism are selected, the acrylate pathway also constitutes a thermodynamically feasible option for propionate production, with lower dependency on CO_2_ availability at circumneutral pH (Eq. 11, Additional file 1: Table S2), as in the case of GG_N2.

Another remarkable result corresponds to the increase in the absolute abundance of the class Negativicutes, particularly the family Veillonellaceae, when glycerol and elevated pCO_2_ were present (GG_CO2 and GLY_CO2, Additional file 1: Fig. S1A). In systems degrading glycerol at circumneutral pH, a concomitant increase in the abundance of Veillonellaceae and Clostridiaceae has been observed [79]. Propionate production is the preferred pathway for glycerol conversion in most members of Veillonellaceae [3, 80, 81], which also became evident in the strong positive correlation between Veillonellaceae and high propionate concentrations (Fig. 6). In this family and the Negativicutes class, the most common pathway for propionate production is the succinate pathway, i.e., phosphoenolpyruvate (PEP) → succinate → propionate, where CO_2_ plays a role. Glycerol conversion experiments with open mixed cultures gave limited emphasis to CO_2_ evolution in the headspace or liquid medium [3, 79]; thus, an indirect causal relation between CO_2_ levels and enhanced propionate production via the selection of members from the Veillonellaceae family, with reported acetogenic nature in the lineage [82], may have been overlooked. However, the observation that Veillonellaceae was a less relevant group in GG_N2 (Fig. 5A), where glycerol was present but not CO_2_, supports the hypothesis of CO_2_ requirement for Veillonellaceae predominance.

The sustained predominance of class Methanomicrobia (genus *Methanosaeta*), particularly in GG_CO2 and GLY_CO2 and to a lower extent in GLU_CO2 (Fig. 5B), also stands out from the presented results. This observation aligns with our previous results [55] and prior pressurized AD studies [26]. Methanosaetaceae became predominant in the archaeal community, although elevated pCO_2_ conventionally results in thermodynamic constraints towards acetotrophic methanogenesis [29]. In addition, *Methanosaeta* is unable to directly utilize hydrogen and formate for CO_2_ reduction to CH_4_—[83]. However, additional thermodynamics analyses showed that increasing acetate concentrations (phase II-C) (Table 2) might compensate for detrimental effects of elevated pCO_2,_ ultimately helping to develop favorable bioenergetics for acetotrophic methanogenesis (Additional file 1: Fig. S3). A recent study by [3] proposed that *Methanosaeta* may be more resilient to increasing carboxylate concentrations, further explaining the predominance of this group during Experiment II.

A possible link between increased pCO_2_ levels and the abundance of members of the class Anaerolinea, whose relative abundance remained high throughout experiment II (Fig. 5A), remains to be elucidated. On one side, since these microorganisms were highly abundant in the original inoculum (Fig. 5A), they could be considered part of the core anaerobic microbiome as other close relatives in the Chloroflexi phylum [84]. On the other side, the observed fluctuations in absolute abundances throughout experiment II (Additional file 1: Fig. S1A) suggest an effect of applied operational conditions on this microbial group. Pressure and higher concentrations of carboxylic acids could have selected for Anaerolinea populations, due to their adhesive feature enabling attachment, formation of protective structures and exchange of intermediate products [85]. Furthermore, recent research in deep-sea sediments has provided evidence of a homoacetogenic lifestyle for members of phylum Chloroflexi [86]. Thus, if elevated pCO_2_ selected for homoacetogens in the Anaerolinea class, this may constitute additional explanatory evidence for the coexistence with *Methanosaeta* spp. as observed in this research (Additional file 1: Fig. S1) and other anaerobic reactors [84, 87]. These findings encourage further exploration of this presumed syntrophic relation in atmospheric and pressurized anaerobic systems.

We have elaborated on the possible roles of microbial groups present after the anaerobic microbiome was exposed to elevated pCO_2_ and to successive changes in the S/X ratio and addition of external electron donor. However, since “presence does not imply activity” [88], the next step in the exploration of the microbial ecology of high-pressure anaerobic digesters will be the application of more advanced -omics (metagenomics, metatranscriptomics) to gain insight into the gene expression and pathway assembly in response to conditions of elevated pCO_2_ [89, 90].

### Combined pH–pCO_2_ effects impact pathway feasibility in anaerobic systems leading to modified product spectrum

Sequential batch operation at elevated pCO_2_, with moderately high substrate concentration, high S/X ratio and relatively short phase durations, led to constrained complete acid conversion (Fig. 3). Acid accumulation, in turn, could have had a more substantial effect due to higher pH fluctuations than anticipated during the provision of 150 mM buffer as HCO_3_^−^. Thus, the system could have experienced lower pH than the calculated value of 6.5, leading to higher levels of undissociated carboxylic acids than those predicted in experiment II and consequently to product-induced inhibition (Fig. 7). Propionate conversion is inhibited by concentrations > 80 mg L^−1^ undissociated propionic acid (HPr) and ≈ 3 mg L^−1^ undissociated acetic acid (HAc) at pH = 7 [91]. Limited propionate conversion at a higher HPr concentration of 260 mg L^−1^ has been reported [35] and coincides with the upper boundary for predicted HPr in experiment II (Fig. 7D). Inhibition of the specific acetic acid utilization rate in methanogens at 145 mg HAc L^−1^ was reported by [92] and a higher concentration of 1141 mg HAc L^−1^ caused inhibitory effects on hydrogen yield from glucose [93]. A 37–60% reduction in the net carboxylates production from glucose at 2000 mg HAc L^−1^, 1400 mg HPr L^−1^ and 120 mg HBu L^−1^, has also been documented [66]. Thus, product-induced inhibition due to increased undissociated carboxylic acids, as an outcome of combined pH–pCO_2_ effects, could help to explain carboxylates (e.g., propionate) accumulation and changes in product spectrum at elevated pCO_2_ and high S/X ratio. However, it cannot be considered the standalone explanatory mechanism in experiment II, since carboxylates accumulation occurred already in phase II-A at lower concentrations of undissociated carboxylic acids (Figs. 2, 7). Therefore, increasing undissociated carboxylic acid concentrations complement the explanatory mechanism for steering product formation in experiment II based on the interaction effects between pCO_2_–process conditions and its modification of microbial community composition.

Total concentrations of carboxylates, particularly undissociated ones, could become a strong modifier of microbial community structure [94] and activity [95]. Hence, in the scenario of increasing undissociated carboxylic acid concentrations in experiment II, dissimilarities in the abundance of syntrophic partners constrained microbial syntrophy. These dissimilarities were associated with a significant, moderate negative correlation between archaeal abundance and undissociated carboxylic acids (Additional file 1: Fig. S2A) and the significant differences in the abundance of syntrophic organisms from class Synergistia (*p* < 0.05) when phase was considered the grouping factor in GG_CO2. Eventually, the constrained syntrophic conversion of propionate and butyrate would have led to the divergence of the pressurized anaerobic system from CH_4_ to carboxylates production in a single reactor without the addition of methanogenic inhibitors due to interaction effects between elevated pCO_2_, pH and high S/X ratio in experiment II. However, more experiments are needed to extrapolate our present observations regarding the steering of product formation from CH_4_ to carboxylates based on the interaction effect of operational parameters. In future experiments, other types of anaerobic inoculum shall be used and a higher variability in the range of selected operational conditions shall be applied to address the issue of reproducibility.

## Methods

### Inoculum

Flocculent anaerobic sludge was obtained from an anaerobic membrane bioreactor (AnMBR), treating wastewater from a food and feed industry, as reported in [36]. The physicochemical characteristics of the inoculum are presented in Table 4. The inoculum was stored for one month at 5.6 °C. After that, biomass was pre-incubated at its original operational temperature (35 °C) and concomitantly activated with 50 mg L^−1^ substrate (glucose) for 24 h before starting the experiments.

**Table 4:** Physicochemical characterization of anaerobic inoculum used in experiments I and II*

Parameter	Unit	Mean ± SD
Total chemical oxygen demand (TCOD)^a^	g L^−1^	30.7 ± 0.2
Soluble COD (SCOD)	mg L^−1^	275 ± 1.2
Total suspended solids (TSS)	g L^−1^	15.7 ± 0.3
Volatile suspended solids (VSS)^a^	g L^−1^	15.2 ± 0.1
VSS/TSS	%	95 ± 2
Ammonium (NH_4_–N)	mg L^−1^	21.7 ± 0.2
Total phosphorous (TP)	mg L^−1^	49.8 ± 3.8
pH	–	7.3

*Average and standard deviations calculated from technical replicates (*n* = 3)

^a^The high COD/VSS ratio is attributed to the presence of fats in the original inoculum, since it was treating influent containing residues from chocolate and animal feed production

### Mixed substrate conversion under elevated pCO_2_

Sequential batch experiments were carried out to investigate the effect of successive changes in operational conditions (e.g., high S/X ratio and addition of formate as external electron donor) on carboxylates and CH_4_ production under elevated pCO_2_ (5 bar). Batch incubation modality was selected based on the premise of anticipated differentiated microbial community responses depending on the way the stress condition is applied (direct vs. successive), as reported for other stressors such as high NH_4_^+^ concentrations [61]. An operational pressure of 5 bar pCO_2_ was selected as a boundary condition between extended lag-phases and noticeable CO_2_ effects on substrate conversion based on previous work [29, 55]. Pressurized stainless-steel reactors (200 mL) (Nantong Vasia, China) were employed for the batch incubations. The liquid medium (120 mL), added to each reactor, consisted of substrate, macro and micronutrients solution prepared according to [96] and buffer solution at a concentration of 150 mM as HCO_3_^−^ to keep pH around 7.5. Concentrations and molar ratios between glycerol and glucose in the feeding solution varied in the different experiments, as explained in Table 5.

**Table 5:** Overview of dual substrate conversion experiments under elevated partial pressure of carbon dioxide (pCO_2_)

Experiment	Description	Conditions			
		Duration (h)	pCO_2_ (bar)	Biomass (g VSS L^−1^)	Soluble COD reactor^a^ (g L^−1^)
I	Reference conversion rate of glucose, glycerol and mixed substrate (1:1 molar ratio) under elevated pCO_2_	7	5	4.4	3.7
II-A	Mixed substrate effect on carboxylates production under elevated pCO_2_ (1:1 molar ratio)	0–216	5	4.4	5
II-B	Effect of higher substrate concentration to increase the substrate-to-biomass ratio (S/X) on carboxylates production under elevated pCO_2_	216–376	5	2.1	10
II-C	Mixed substrate effect on carboxylates production under elevated pCO_2_ (1:1 molar ratio) + additional electron donor (formate—5 mM)	376–596	5	2.0	5

^a^COD reactor corresponds to the intended concentration after feeding concentrated substrate solution to each reactor

#### Experiment I: determination of reference substrate conversion rate under elevated pCO_2_

This experiment was conducted to estimate the conversion rates of glycerol, glucose and the mixture (1:1 molar ratio) at 5 bar pCO_2_. We used the activated and pre-incubated inoculum described in the previous section and the operational conditions summarized in Table 5.

#### Experiment II: mixed substrate (glycerol and glucose) conversion under elevated pCO_2_

This experiment consisted of three sequential phases (II-A, II-B and II-C) with varying operational conditions under elevated pCO_2_ to monitor shifts in product spectrum and microbial community structure. Experiments corresponding to the main condition of interest, mixed substrate of glycerol and glucose (GG_CO2), were carried out in triplicates. Three single controls were included for individual glycerol and glucose conversion at 5 bar pCO_2_ (GLY_CO2, GLU_CO2) and conditions of pressurized headspace with a non-reactive gas, i.e., 5 bar using nitrogen (GG_N2). Due to limited reactor availability, controls were carried out in parallel as single reactors. This approach was chosen to avoid differences in the characteristics of the starting inoculum between the main condition and controls if otherwise decided to carry out triplicate controls as temporal sequential batches. Stainless steel reactors were inoculated with acclimated inoculum, incubated at 35 °C and continuously shaken at 110 rpm. Samples (2 mL liquid and 10 mL gas) were taken trice per day (first 2 days), twice per day (subsequent days) and once per day (last 3–4 days) to measure substrate conversion and formation of liquid and gaseous products in each phase. From liquid samples collected at the initial (t = 0 h) and the endpoint of each phase, 250 µL was fixed with glutaraldehyde (1% v/v) and stored at 5 °C for total cell determination by flow cytometry.

#### Phase II-A: mixed substrate conversion

Operational conditions and experiment duration are reported in Table 5. Macro- and micronutrients were proportionally dosed according to the increase in initial COD to prevent nutrient limitations. Buffer solution (150 mM as HCO_3_^−^) was provided in the feeding solution. Reactor headspace was adjusted to 5 bar pCO_2_ following the methodology described in [29] and left for equilibration with the liquid phase for 2 h. The experiment was terminated after complete substrate depletion and > 70% soluble COD was recovered in liquid and gaseous products.

#### Phase II-B: effect of high substrate concentration to increase S/X ratio

After the final liquid and gas sampling in experiment II-A, 20 mL was removed from all reactors via the liquid sampling port and replaced by fresh medium (40 mL in total) to start phase II-B at a moderate volumetric exchange ratio of 33%. Fresh medium for reactors GG_CO2 consisted of a concentrated solution to achieve substrate concentrations indicated in Table 5. Macro- and micronutrients were proportionally dosed. The fresh medium was injected into the pressurized reactors employing a pressure-resistant, stainless steel, double-ended liquid sampling vessel with an effective volume of 100 mL (Swagelok, US). One side of the vessel was connected to a > 99% compressed CO_2_ bottle, set at 2 bar overpressure from the manometer reading. The other side was connected to one of the liquid sampling ports of the pressure reactors controlled by a stainless-steel needle valve. Pressure deviations occurred after liquid extraction and new medium injection; thus, before restarting the experiment, headspace pressure was adjusted to 5 bar total pressure with > 99% CO_2_ or N_2_. After one hour stabilization period, gas samples (10 mL) were taken to determine the initial gas composition. Experiment II-B was finalized after complete substrate depletion (10 days comparable with phase II-A), corresponding to a COD-recovery > 50% in gaseous and liquid products.

#### Phase II-C: effect of external electron donor (formate)

This experiment was initiated after final liquid and gas sampling in phase II-B. Reactor feeding and re-pressurization were carried out as previously described for phase II-B and under the operational conditions mentioned in Table 5. Formate (5 mM) was added to the concentrated feeding solution to evaluate the effect of additional electron donor in the product spectrum under elevated pCO_2_.

### Analyses

#### Physicochemical analyses

Secondary metabolites, i.e., acetate, propionate, butyrate and valerate were measured from filtered (0.45 μm) liquid samples by gas chromatography (7890A GC, Agilent Technologies, US) according to [97]. The detection limits for these compounds were 12, 16, 18 and 23 mg L^−1^, respectively. The method and device settings also allowed alcohol determination (ethanol, propanol, butanol); however, amounts in our samples were below the detection limits (5, 2.5 and 2.5 mg L^−1^, respectively). Glucose, glycerol, formate, succinate and lactate were measured in filtered (0.45 μm), acidified samples by high-performance liquid chromatography (LC-20AT; Shimadzu, Japan) using an Aminex HPX-87H (300 × 7.8 mm) column with sulphuric acid (5 mM) as eluent. Operational conditions were as follows: flow rate of 0.5 mL min^−1^, RID-20A detector at 50 °C for glucose and glycerol determination and SPD-20A detector at 40 °C with wavelength at 210 nm for organic acids. The detection limits were 50 mg L^−1^ for organic acids, glucose, and glycerol according to prepared calibration curves. Gas samples (10 mL) were measured by gas chromatography (7890A GC, Agilent Technologies, US) as described in [55]. The pH (inoLab® Multi 9620 IDS), total and soluble COD, TSS and VSS, ammonium and total phosphorus were measured according to standard methods [98].

#### Total cell numbers

Total cell numbers were assessed by flow cytometry (AttuneTM NxT 2019; InvitrogenTM—ThermoFisher SCIENTIFIC, US) using Mili-Q as sheath fluid. Pre-treatment started with fixed samples vortexed and diluted (1:10) with 0.20-μm filtered phosphate-buffered saline (PBS) solution. Next, diluted samples were sonicated (100 W) for 3 min at room temperature, vortexed, filtered at 20 µm with falcon, sterile, syringe-type filters (BD BIOSCIENCES, US) and serially diluted (1:1000). After pre-treatment, samples were placed in 96-well plates, stained with 5% SYBR^®^ Green I (InvitrogenTM—ThermoFisher SCIENTIFIC, US) and incubated at 37 °C for 20 min. The AttuneTM NxT 2019 was used in the BRxx configuration with two lasers: 480 nm and 635 nm. The channel used during the measurements corresponded to BL1 (530/30).

#### Microbial community analysis and statistical processing

Liquid samples (1.5 mL) were centrifuged at 12,298 RCF for 2 min and obtained biomass pellets were collected and stored at − 80 °C. The DNA extraction was done according to the DNeasy UltraClean Microbial Kit (Qiagen, Germany). The DNA quality and quantity were controlled using Qubit® 3.0 DNA detection (Qubit dsDNA HS Assay Kit, Life Technologies, United States). Library construction, sequencing in the Illumina platform and preliminary data processing were done according to the internal protocol from Novogene (Hong Kong) (Additional file 1: Materials and methods). Statistical analyses from microbial community data were carried out in R version 3.6.1 (http://www.r-project.org) [99]. Canonical correspondence analysis (CCA) was performed in R software [99], employing the function *cca* from the vegan package [100]. The CCA was selected for the analysis, since it effectively assesses how environmental factors or process conditions (pCO_2_, carboxylates concentration, formate) relate to the microbial community structure [101]. The significance of the ordination based on the selected environmental constraints and of the canonical axes was tested via permutation analysis (*anova.cca*). As a measurement of alpha diversity, community richness was calculated based on the total number of taxa after singleton removal, using the function *estimate_richness()* from the phyloseq package [102]. Significant differences (p < 0.05) in beta diversity, calculated using the Bray–Curtis distance measure [103], were identified employing pairwise permutational ANOVA (PERMANOVA) using the *adonis* function (vegan). Spearman’s correlation analyses were carried out using the function *cor.test ()*. Non-parametric analysis of variance was performed using the Wilcoxon test for paired samples. The variance in carboxylates concentration (acetate, propionate, butyrate), succinate concentration and cell density, due to the main effects and interactions of three independent factors, e.g., process conditions as gas pressure, substrate concentration and additional electron donor was analyzed with the R package ARTool (Align-and-rank data for non-parametric factorial ANOVA) [104].

#### Estimation of substrate conversion rates

The application “Simple fit” from OriginPro [105] was used to adjust non-linear or linear models to describe substrate conversion. Logistic models and linear regression have been previously reported in the literature as good approximations to describe soluble substrate utilization in AD [106, 107]. Simple linear regression and the logistic model were employed to fit the data in experiment I, whereas only the logistic model proved adequate to fit the data in experiment II.

The logistic model equation (Eq. 1) corresponds to

1 $$y=\frac{a}{\left(1+\mathrm{exp}\left(-k\left(x-{x}_{\text{C}}\right)\right)\right)},$$

where *y* represents the substrate concentration, *a* the maximal initial substrate concentration (mg L^−1^), *k* is the logistic model constant comparable to the consumption rate (h^−1^), *x* corresponds to time (h) and *x*_c_ is the time point where the sigmoid changes its curvature.

### Calculations

#### Bioenergetics

Thermodynamic calculations were developed according to [108] to establish the energy feasibility of biochemical reactions possibly occurring in pressurized experiments. Substrate and product concentrations corresponded to the physiological range (1 mM) and corrections were applied only for mesophilic temperature (35 °C) and initial pH (7.5).

#### Reactor pH under elevated pCO_2_ and concentration of undissociated carboxylic acids

Due to limitations in the employed experimental set-up, the pH could not be continuously registered during the pressurized experiments. Therefore, we estimated the lowest equilibrium pH possibly achieved in the system after the equilibration of CO_2_ concentrations between the headspace and the liquid medium based on Henry’s law (Eq. 2) and the Henderson–Hasselbalch equation (Eq. 3). This pH value was used to calculate the expected undissociated carboxylic acid concentrations throughout experiment II, based on the GC measurements for acetate, propionate and butyrate. Equilibrium constants (*k*_H_, *K*_a_) were corrected for mesophilic temperature (35 °C). The p*Ka* values for acetic, propionic and butyric were obtained from [66] and for H_2_CO_3_* from [109]. For the Spearman correlation analysis, the total concentration of undissociated acids was expressed in acetic acid equivalents (mg L^−1^) according to the method described by [110]:

2 $${\text{H}}_{2}\text{CO}_{3}^{*}=\text{p}_{\text{CO}_{2}}*{K}_{H},$$

3 $${\text{pH}}=\text{p}K_{a}+log\frac{ [{A}^{-}]}{[HA]}.$$

## Conclusions

Elevated pCO_2_ can act as a steering parameter in anaerobic processes, e.g., mixed culture fermentation and AD; however, its “*modus operandi*” is complex, and the obtained selectivity leads to trade-offs with substrate consumption rates and lag-phases. Changes in product spectrum and cell density, rather than an isolated effect of increasing pCO_2,_ showed dependency on the interaction effects with process conditions, such as S/X ratio and availability of external electron donor. Interaction effects between elevated pCO_2,_ substrate specificity and the aforementioned process conditions modified the microbial community composition, e.g., higher abundance of acetogenic microorganisms, whose activity further modified the product spectrum. Succinate production was observed as a result of the interaction effect between pCO_2_ and formate in mixed substrate experiments after subsequent pressurized cycles. Succinate production was attributed to the availability of extra reducing equivalents, likely enhanced carbon fixating activity and end-product accumulation (propionate) due to increasing concentrations of undissociated carboxylic acids.

## Supplementary Information

**Additional file 1:** Supplementary Methods, Calculations and Results

## Data Availability

The raw fastq files used to create the OTU table for the microbial community analysis, have been deposited in the National Center for Biotechnology Information (NCBI) database (Accession number PRJNA704781).

## Abbreviations

ABE: Acetone–butanol–ethanol
AD: Anaerobic digestion
CCA: Canonical correspondence analysis
COD: Chemical oxygen demand
EoP: End-of-phase
FCM: Flow cytometry measurement
HAc: Undissociated acetic acid
HBu: Undissociated butyric acid
HPAD: High-pressure anaerobic digestion
HPr: Undissociated propionic acid
NA: Not available
pCO_2_: CO_2_ partial pressure
PEP: Phosphoenolpyruvate
SCOD: Soluble chemical oxygen demand
TCOD: Total chemical oxygen demand
TP: Total phosphorous
TSS: Total suspended solids
VSS: Volatile suspended solids
WLP: Wood–Ljungdahl pathway

## References

[1] Cherubini F (2010). The biorefinery concept: using biomass instead of oil for producing energy and chemicals. Energy Convers Manag.

[2] Marshall CW, LaBelle EV, May HD (2013). Production of fuels and chemicals from waste by microbiomes. Curr Opin Biotechnol.

[3] Braz GHR, Fernandez-Gonzalez N, Lema JM, Carballa M (2019). Organic overloading affects the microbial interactions during anaerobic digestion in sewage sludge reactors. Chemosphere.

[4] Agler MT, Wrenn BA, Zinder SH, Angenent LT (2011). Waste to bioproduct conversion with undefined mixed cultures: the carboxylate platform. Trends Biotechnol.

[5] Rodríguez J, Kleerebezem R, Lema JM, van Loosdrecht MCMM (2006). Modeling product formation in anaerobic mixed culture fermentations. Biotechnol Bioeng.

[6] Kleerebezem R, van Loosdrecht MCM (2007). Mixed culture biotechnology for bioenergy production. Curr Opin Biotechnol.

[7] Angenent LT, Wrenn BA. Optimizing mixed-culture bioprocessing to convert wastes into bioenergy. In: Bioenergy. 2008. p. 179–94.

[8] Carballa M, Regueiro L, Lema JM (2015). Microbial management of anaerobic digestion: exploiting the microbiome-functionality nexus. Curr Opin Biotechnol.

[9] Jankowska E, Chwialkowska J, Stodolny M, Oleskowicz-Popiel P (2017). Volatile fatty acids production during mixed culture fermentation—the impact of substrate complexity and pH. Chem Eng J.

[10] Coma M, Vilchez-Vargas R, Roume H, Jauregui R, Pieper DH, Rabaey K (2016). Product diversity linked to substrate usage in chain elongation by mixed-culture fermentation. Environ Sci Technol.

[11] Hoelzle RD, Puyol D, Virdis B, Batstone D (2021). Substrate availability drives mixed culture fermentation of glucose to lactate at steady state. Biotechnol Bioeng.

[12] Zoetemeyer RJJ, van den Heuvel JCC, Cohen A (1982). pH influence on acidogenic dissimilation of glucose in an anaerobic digester. Water Res Pergamon.

[13] Tamis J, Joosse BMM, van Loosdrecht MCM, Kleerebezem R, Loosdrecht MCM, Kleerebezem R (2015). High-rate volatile fatty acid (VFA) production by a granular sludge process at low pH. Biotechnol Bioeng.

[14] Temudo MF, Kleerebezem R, van Loosdrecht M (2007). Influence of the pH on (open) mixed culture fermentation of glucose: a chemostat study. Biotechnol Bioeng.

[15] Lee M, Hidaka T, Tsuno H (2008). Effect of temperature on performance and microbial diversity in hyperthermophilic digester system fed with kitchen garbage. Bioresour Technol.

[16] Zhuo G, Yan Y, Tan X, Dai X, Zhou Q (2012). Ultrasonic-pretreated waste activated sludge hydrolysis and volatile fatty acid accumulation under alkaline conditions: effect of temperature. J Biotechnol.

[17] Khan MAA, Ngo HHH, Guo WSS, Liu Y, Nghiem LDD, Hai FII (2016). Optimization of process parameters for production of volatile fatty acid, biohydrogen and methane from anaerobic digestion. Bioresour Technol.

[18] Dai K, Wen J-L, Zhang F, Zeng RJ (2017). Valuable biochemical production in mixed culture fermentation: fundamentals and process coupling. Appl Microbiol Biotechnol.

[19] Lim SJ, Kim BJ, Jeong CM, Ahn YH, Chang HN (2008). Anaerobic organic acid production of food waste in once-a-day feeding and drawing-off bioreactor. Bioresour Technol.

[20] Jiang J, Zhang Y, Li K, Wang Q, Gong C, Li M (2013). Volatile fatty acids production from food waste: effects of pH, temperature, and organic loading rate. Bioresour Technol.

[21] Arslan D, Steinbusch KJJJJ, Diels L, De Wever H, Buisman CJNJN, Hamelers HVMVM (2012). Effect of hydrogen and carbon dioxide on carboxylic acids patterns in mixed culture fermentation. Bioresour Technol.

[22] Arslan D, Steinbusch KJJ, Diels L, De Wever H, Hamelers HVM, Buisman CJN (2013). Selective carboxylate production by controlling hydrogen, carbon dioxide and substrate concentrations in mixed culture fermentation. Bioresour Technol.

[23] De Kok S, Meijer J, Van Loosdrecht MCMM, Kleerebezem R (2013). Impact of dissolved hydrogen partial pressure on mixed culture fermentations. Appl Microbiol Biotechnol.

[24] Lindeboom REF, Fermoso FG, Weijma J, Zagt K, van Lier JB (2011). Autogenerative high pressure digestion: anaerobic digestion and biogas upgrading in a single step reactor system. Water Sci Technol.

[25] Bothun GDD, Knutson BLL, Berberich JAA, Strobel HJJ, Nokes SEE (2004). Metabolic selectivity and growth of *Clostridium thermocellum* in continuous culture under elevated hydrostatic pressure. Appl Microbiol Biotechnol.

[26] Lindeboom REF, Shin SG, Weijma J, van Lier JB, Plugge CM (2016). Piezo-tolerant natural gas-producing microbes under accumulating pCO_2_. Biotechnol Biofuels.

[27] Zhao J, Li Y, Marandola C, Krooneman J, Euverink GJW (2020). Comparison of the microbial communities in anaerobic digesters treating high alkalinity synthetic wastewater at atmospheric and high-pressure (11 bar). Bioresour Technol.

[28] Wan R, Chen Y, Zheng X, Su Y, Huang H (2018). Effect of CO_2_ on NADH production of denitrifying microbes via inhibiting carbon source transport and its metabolism. Sci Total Environ.

[29] Ceron-Chafla P, Kleerebezem R, Rabaey K, van Lier JB, Lindeboom RE (2020). Direct and indirect effects of increased CO_2_ partial pressure on the bioenergetics of syntrophic propionate and butyrate conversion. Environ Sci Technol.

[30] Girbal L, Soucaille P (1994). Regulation of *Clostridium acetobutylicum* metabolism as revealed by mixed- substrate steady-state continuous cultures: role of NADH/NAD ratio and ATP pool. J Bacteriol.

[31] Berríos-Rivera SJ, Bennett GN, San KY (2002). The effect of increasing NADH availability on the redistribution of metabolic fluxes in *Escherichia coli* chemostat cultures. Metab Eng.

[32] Saint-Amans S, Girbal L, Andrade J, Ahrens K, Soucaille P (2001). Regulation of carbon and electron flow in *Clostridium butyricum* VPI 3266 grown on glucose-glycerol mixtures. J Bacteriol.

[33] Vasconcelos I, Girbal L, Soucaille P (1994). Regulation of carbon and electron flow in *Clostridium acetobutylicum* grown in chemostat culture at neutral pH on mixtures of glucose and glycerol. J Bacteriol.

[34] Hakobyan B, Pinske C, Sawers G, Trchounian A, Trchounian K (2018). PH and a mixed carbon-substrate spectrum influence FocA- and FocB-dependent, formate-driven H_2_ production in *Escherichia coli*. FEMS Microbiol Lett.

[35] Mösche M, Jördening HJ (1999). Comparison of different models of substrate and product inhibition in anaerobic digestion. Water Res.

[36] Ceron-Chafla P, Chang Y-T, Rabaey K, van Lier JB, Lindeboom REF (2021). Directional selection of microbial community reduces propionate accumulation in glycerol and glucose mixed culture fermentations under elevated pCO_2_. Front Microbiol.

[37] Amulya K, Mohan SV (2019). Fixation of CO_2_, electron donor and redox microenvironment regulate succinic acid production in Citrobacter amalonaticus. Sci Total Environ.

[38] Sawers RG, Clark DP. Fermentative pyruvate and acetyl-coenzyme a metabolism. EcoSal Plus. 2004;1. 10.1128/ecosalplus.3.5.3 26443368

[39] Song H, Lee JW, Choi S, You JK, Hong WH, Lee SY (2007). Effects of dissolved CO_2_ levels on the growth of *Mannheimia*
*succiniciproducens* and succinic acid production. Biotechnol Bioeng.

[40] Sauer U, Eikmanns BJ (2005). The PEP-pyruvate-oxaloacetate node as the switch point for carbon flux distribution in bacteria. FEMS Microbiol Rev.

[41] Ragsdale SW, Pierce E (2008). Acetogenesis and the Wood-Ljungdahl pathway of CO_2_ fixation. Biochim Biophys Acta Proteins Proteomics.

[42] Jones SW, Fast AG, Carlson ED, Wiedel CA, Au J, Antoniewicz MR (2016). CO_2_ fixation by anaerobic non-photosynthetic mixotrophy for improved carbon conversion. Nat Commun.

[43] Fast AG, Schmidt ED, Jones SW, Tracy BP (2015). Acetogenic mixotrophy: novel options for yield improvement in biofuels and biochemicals production. Curr Opin Biotechnol.

[44] Maru BT, Munasinghe PC, Gilary H, Jones SW, Tracy BP (2018). Fixation of CO_2_ and CO on a diverse range of carbohydrates using anaerobic, non-photosynthetic mixotrophy. FEMS Microbiol Lett.

[45] Angenent LT, Richter H, Buckel W, Spirito CM, Steinbusch KJJ, Plugge CM (2016). Chain elongation with reactor microbiomes: open-culture biotechnology to produce biochemicals. Environ Sci Technol.

[46] Wainaina S, Lukitawesa, Kumar Awasthi M, Taherzadeh MJ (2019). Bioengineering of anaerobic digestion for volatile fatty acids, hydrogen or methane production: a critical review. Bioengineered.

[47] Heffernan JK, Valgepea K, de Souza Pinto Lemgruber R, Casini I, Plan M, Tappel R (2020). Enhancing CO_2_-valorization using *Clostridium autoethanogenum* for sustainable fuel and chemicals production. Front Bioeng Biotechnol.

[48] Conrad R (2020). Importance of hydrogenotrophic, aceticlastic and methylotrophic methanogenesis for methane production in terrestrial, aquatic and other anoxic environments: a mini review. Pedosphere.

[49] Oppermann BI, Michaelis W, Blumenberg M, Frerichs J, Schulz HM, Schippers A (2010). Soil microbial community changes as a result of long-term exposure to a natural CO_2_ vent. Geochim Cosmochim Acta.

[50] Ziels RM, Nobu MK, Sousa DZ (2019). Elucidating syntrophic butyrate-degrading populations in anaerobic digesters using stable-isotope-informed genome-resolved metagenomics. mSystems..

[51] Li L, He Q, Ma Y, Wang X, Peng X (2016). A mesophilic anaerobic digester for treating food waste: process stability and microbial community analysis using pyrosequencing. Microb Cell Fact.

[52] Liu Y, Zhang YG, Zhang RB, Zhang F, Zhu J (2011). Glycerol/glucose co-fermentation: one more proficient process to produce propionic acid by Propionibacterium acidipropionici. Curr Microbiol.

[53] Wang Z, Yang S-T (2013). Propionic acid production in glycerol/glucose co-fermentation by *Propionibacterium freudenreichii* subsp. shermanii. Bioresour Technol.

[54] Props R, Kerckhof FM, Rubbens P, De VJ, Sanabria EH, Waegeman W (2017). Absolute quantification of microbial taxon abundances. ISME J.

[55] Ceron-Chafla P, Chang Y, Rabaey K, van Lier JB, Lindeboom REF (2021). Directional selection of microbial community reduces propionate accumulation in glycerol and glucose anaerobic bioconversion under elevated pCO_2_. Front Microbiol.

[56] Stams AJM, Plugge CM (2009). Electron transfer in syntrophic communities of anaerobic bacteria and archaea. Nat Rev Microbiol.

[57] Dolfing J, Jiang B, Henstra AM, Stams AJM, Plugge CM (2008). Syntrophic growth on formate: a new microbial niche in anoxic environments. Appl Environ Microbiol.

[58] Ramió-Pujol S, Ganigué R, Bañeras L, Colprim J (2015). Impact of formate on the growth and productivity of *Clostridium ljungdahlii* PETC and *Clostridium carboxidivorans* P7 grown on syngas. Int Microbiol.

[59] Asanuma N, Iwamoto M, Hino T (1999). Effect of the addition of fumarate on methane production by ruminal microorganisms in vitro. J Dairy Sci.

[60] Stams AJM, Grolle KCF, Frijters CTMJ, Van Lier JB (1992). Enrichment of thermophilic propionate-oxidizing bacteria in syntrophy with *Methanobacterium thermoautotrophicum* or *Methanobacterium thermoformicicum*. Appl Environ Microbiol.

[61] Hardy J, Bonin P, Lazuka A, Gonidec E, Guasco S, Valette C (2021). Similar methanogenic shift but divergent syntrophic partners in anaerobic digesters exposed to direct versus successive ammonium additions. Microbiol Spectr.

[62] Parizzi LP, Grassi MCB, Llerena LA, Carazzolle MF, Queiroz VL, Lunardi I (2012). The genome sequence of *Propionibacterium acidipropionici* provides insights into its biotechnological and industrial potential. BMC Genomics.

[63] Zhang A, Yang S-T (2009). Propionic acid production from glycerol by metabolically engineered *Propionibacterium acidipropionici*. Process Biochem.

[64] Tan Z, Zhu X, Chen J, Li Q, Zhanga X (2013). Activating phosphoenolpyruvate carboxylase and phosphoenolpyruvate carboxykinase in combination for improvement of succinate production. Appl Environ Microbiol.

[65] van Lingen HJ, Plugge CM, Fadel JG, Kebreab E, Bannink A, Dijkstra J (2016). Thermodynamic driving force of hydrogen on rumen microbial metabolism: a theoretical investigation. PLoS ONE.

[66] Xiao K, Zhou Y, Guo C, Maspolim Y, Ng WJ (2016). Impact of undissociated volatile fatty acids on acidogenesis in a two-phase anaerobic system. J Environ Sci.

[67] Lu S, Eiteman MA, Altman E (2009). Effect of CO_2_ on succinate production in dual-phase *Escherichia coli* fermentations. J Biotechnol.

[68] Van Der Werf MJ, Guettler MV, Jain MK, Zeikus JG (1997). Environmental and physiological factors affecting the succinate product ratio during carbohydrate fermentation by *Actinobacillus* sp. 130Z. Arch Microbiol.

[69] Lindeboom REF, Ding L, Weijma J, Plugge CM, van Lier JB (2014). Starch hydrolysis in autogenerative high pressure digestion: gelatinisation and saccharification as rate limiting steps. Biomass Bioenergy.

[70] Kakuk B, Wirth R, Maróti G, Szuhaj M, Rakhely G, Laczi K (2021). Early response of methanogenic archaea to H_2_ as evaluated by metagenomics and metatranscriptomics. Microb Cell Fact.

[71] Pietrocola F, Galluzzi L, Bravo-San Pedro JM, Madeo F, Kroemer G (2015). Acetyl coenzyme A: a central metabolite and second messenger. Cell Metab.

[72] Foladori P, Bruni L, Tamburini S, Ziglio G (2010). Direct quantification of bacterial biomass in influent, effluent and activated sludge of wastewater treatment plants by using flow cytometry. Water Res.

[73] Emerson JE, Stabler RA, Wren BW, Fairweather NF (2008). Microarray analysis of the transcriptional responses of *Clostridium difficile* to environmental and antibiotic stress. J Med Microbiol.

[74] Jain S, Dietrich HM, Müller V, Basen M (2020). Formate is required for growth of the thermophilic acetogenic bacterium *Thermoanaerobacter*
*kivui* lacking hydrogen-dependent carbon dioxide reductase (HDCR). Front Microbiol.

[75] Patakova P, Linhova M, Rychtera M, Paulova L, Melzoch K (2013). Novel and neglected issues of acetone–butanol–ethanol (ABE) fermentation by clostridia: Clostridium metabolic diversity, tools for process mapping and continuous fermentation systems. Biotechnol Adv.

[76] Kleerebezem R, Joosse B, Rozendal R, Van Loosdrecht MCM (2015). Anaerobic digestion without biogas?. Rev Environ Sci Bio/Technol.

[77] Gonzalez-Garcia RA, McCubbin T, Navone L, Stowers C, Nielsen LK, Marcellin E (2017). Microbial propionic acid production. Fermentation.

[78] Barbirato F, Chedaille D, Bories A (1997). Propionic acid fermentation from glycerol: comparison with conventional substrates. Appl Microbiol Biotechnol.

[79] Moscoviz R, Trably E, Bernet N (2016). Consistent 1,3-propanediol production from glycerol in mixed culture fermentation over a wide range of pH. Biotechnol Biofuels.

[80] Ferguson RMW, Coulon F, Villa R (2018). Understanding microbial ecology can help improve biogas production in AD. Sci Total Environ.

[81] Xafenias N, Anunobi MSO, Mapelli V (2015). Electrochemical startup increases 1,3-propanediol titers in mixed-culture glycerol fermentations. Process Biochem.

[82] Aryal N, Tremblay PL, Lizak DM, Zhang T (2017). Performance of different Sporomusa species for the microbial electrosynthesis of acetate from carbon dioxide. Bioresour Technol.

[83] Rotaru AE, Shrestha PM, Liu F, Shrestha M, Shrestha D, Embree M (2014). A new model for electron flow during anaerobic digestion: direct interspecies electron transfer to Methanosaeta for the reduction of carbon dioxide to methane. Energy Environ Sci.

[84] Bovio-Winkler P, Cabezas A, Etchebehere C (2021). Database mining to unravel the ecology of the Phylum Chloroflexi in methanogenic full scale bioreactors. Front Microbiol.

[85] Xia Y, Wang Y, Wang Y, Chin FYL, Zhang T (2016). Cellular adhesiveness and cellulolytic capacity in Anaerolineae revealed by omics-based genome interpretation. Biotechnol Biofuels.

[86] Sewell HL, Kaster A-K, Sporman AM (2017). Homoacetogenesis in deep-sea Chloroflexi, as inferred by single-cell genomics, provides a link to reductive dehalogenation in terrestrial Dehalococcoidetes. MBio.

[87] McIlroy SJ, Kirkegaard RH, Dueholm MS, Fernando E, Karst SM, Albertsen M (2017). Culture-independent analyses reveal novel anaerolineaceae as abundant primary fermenters in anaerobic digesters treating waste activated sludge. Front Microbiol.

[88] De Vrieze J, Regueiro L, Props R, Vilchez-Vargas R, Jáuregui R, Pieper DH (2016). Presence does not imply activity: DNA and RNA patterns differ in response to salt perturbation in anaerobic digestion. Biotechnol Biofuels.

[89] Wirth R, Pap B, Dudits D, Kakuk B, Bagi Z, Shetty P (2021). Genome-centric investigation of anaerobic digestion using sustainable second and third generation substrates. J Biotechnol.

[90] Treu L, Campanaro S, Kougias PG, Sartori C, Bassani I, Angelidaki I (2018). Hydrogen-fueled microbial pathways in biogas upgrading systems revealed by genome-centric metagenomics. Front Microbiol.

[91] Fukuzaki S, Nishio N, Shobayashi M, Nagai S (1990). Inhibition of the fermentation of propionate to methane by hydrogen, acetate, and propionate. Appl Environ Microbiol.

[92] Xiao KK, Guo CH, Zhou Y, Maspolim Y, Wang JY, Ng WJ (2013). Acetic acid inhibition on methanogens in a two-phase anaerobic process. Biochem Eng J.

[93] Van Ginkel S, Logan BE (2005). Inhibition of biohydrogen production by undissociated acetic and butyric acids. Environ Sci Technol.

[94] Jia X, Wang Y, Ren L, Li M, Tang R, Jiang Y (2019). Early warning indicators and microbial community dynamics during unstable stages of continuous hydrogen production from food wastes by thermophilic dark fermentation. Int J Hydrogen Energy.

[95] Lee HS, Salerno MB, Rittmann BE (2008). Thermodynamic evaluation on H_2_ production in glucose fermentation. Environ Sci Technol.

[96] García Rea VS, Muñoz Sierra JD, Fonseca Aponte LM, Cerqueda-Garcia D, Quchani KM, Spanjers H (2020). Enhancing phenol conversion rates in saline anaerobic membrane bioreactor using acetate and butyrate as additional carbon and energy sources. Front Microbiol.

[97] Muñoz Sierra JD, García Rea VS, Cerqueda-García D, Spanjers H, van Lier JB (2020). Anaerobic conversion of saline phenol-containing wastewater under thermophilic conditions in a membrane bioreactor. Front Bioeng Biotechnol.

[98] American Public Health Association (2017). Standard methods for the examination of water and wastewater.

[99] R Core Team (2019). R: A language and environment for statistical computing.

[100] Oksanen J, Blanchet FG, Kindt R, Legendre P, Minchin PR, O’hara RB, et al. Vegan: community ecology package. R package version 2.3-4. 2016.

[101] Ma H, Liu H, Zhang L, Yang M, Fu B, Liu H (2017). Novel insight into the relationship between organic substrate composition and volatile fatty acids distribution in acidogenic co-fermentation. Biotechnol Biofuels.

[102] McMurdie PJ, Holmes S (2013). phyloseq: an R package for reproducible interactive analysis and graphics of microbiome census data. PLoS ONE.

[103] Bray JR, Curtis JT (1957). An ordination of the upland forest communities of southern Wisconsin. Ecol Monogr Wiley.

[104] Kay M, Elkin L, Higgins J, Wobbrock J. ARTool: aligned rank transform for nonparametric factorial ANOVAs. 2021.

[105] OriginLab Corporation (2019). OriginPro.

[106] Mawson AJ, Earle RL, Larsen VF (1991). Degradation of acetic and propionic acids in the methane fermentation. Water Res.

[107] Coelho MMH, Morais NWS, Pereira EL, Leitão RC, dos Santos AB (2020). Potential assessment and kinetic modeling of carboxylic acids production using dairy wastewater as substrate. Biochem Eng J.

[108] Heijnen JJ, Kleerebezem RR (2010). Bioenergetics of microbial growth. Encycl Ind Biotechnol.

[109] Stumm W, Morgan JJ. Aquatic chemistry: chemical equilibria and rates in natural waters. 3rd ed. Environ. Sci. Technol. New York: Wiley; 1996.

[110] Fu Z, Holtzapple MT (2010). Fermentation of sugarcane bagasse and chicken manure to calcium carboxylates under thermophilic conditions. Appl Biochem Biotechnol.

