# Supplementary material for: Steering the product spectrum in high-pressure anaerobic processes: CO2 partial pressure as a novel tool in biorefinery concepts
Source: Biotechnol Biofuels Bioprod. 2023 Feb 18;16:27. doi: 10.1186/s13068-023-02262-x (PMC9938588; doi:10.1186/s13068-023-02262-x)
Supplement: Supplementary file 1 — Additional file 1: Supplementary Methods, Calculations and Results [file 13068_2023_2262_MOESM1_ESM.docx]

**Steering the product spectrum in high-pressure anaerobic processes: CO_2_ partial pressure as a novel tool in bio-refinery concepts.**

Pamela Ceron-Chafla^1*^, Jo de Vrieze^2^, Korneel Rabaey^2,3^, Jules B. van Lier^1^, Ralph E.F. Lindeboom^1^

^1^ Sanitary Engineering section, Department of Water Management, Delft University of Technology, Stevinweg 1, 2628 CN, Delft, The Netherlands.

^2^ Center for Microbial Ecology and Technology (CMET), Ghent University, Coupure Links 653, B-9000 Ghent, Belgium

^3^ Center for Advanced Process Technology for Urban Resource Recovery (CAPTURE), Coupure Links 653, B-9000 Ghent, Belgium

*Corresponding author: p.s.ceronchafla@tudelft.nl

Number of tables: 2

Number of figures: 3

Number of pages: 7

**Additional Materials and Methods**

## Internal protocol for Illumina sequencing Novogene

During the amplification, DNA concentration and purity were first monitored on 1% agarose gels and diluted to 1ng/μL by sterile water. Then, 16S rRNA genes of distinct regions (16SV3-V4) were amplified with the specific primer (e.g. 16S V4: 515F-806R). The chosen PCR products, between 400 to 450 bp, were mixed in equidensity ratios. Then, the mixture PCR products were purified with Qiagen Gel Extraction Kit (Qiagen, Germany). The libraries of the samples, generated with NEBNext® UltraTM DNA Library Prep Kit for Illumina and quantified via Qubit and Q-PCR, were analyzed by the Illumina platform.

Paired-end reads were assigned to samples based on their unique barcode and truncated by cutting off the barcode and primer sequence. Paired-end reads were merged by FLASH [1], and quality filtering on the raw tags was performed under specific filtering conditions to obtain the high-quality clean tags [2] with the Qiime quality-controlled process [3]. The effective tags were obtained after comparison with UCHIME algorithm [4] the reference database, to detect chimera sequences and subsequent removal of those.

Sequences analysis was performed by Uparse software [5], using all the effective tags. Sequences with ≥97% similarity were assigned to the same operational taxonomic units (OTUs). The representative sequence for each OTU was screened for further annotation. For each representative sequence, Mothur software was performed against the SSUrRNA database of SILVA Database [6] for species annotation at each taxonomic rank (Threshold:0.8~1) [7] (kingdom, phylum, class, order, family, genus, species). To get the phylogenetic relationship of all OTUs representative sequences, the MUSCLE algorithm [8] was applied to compare multiple sequences. OTUs abundance information was normalized using a standard of sequence number corresponding to the sample with the least sequences.

**Additional Tables and figures**

**Table S1:** Relative abundance of total Bacteria and Archaea for the different experimental conditions in Experiment II. Values for treatment mixed substrate treatment (GG_CO2) correspond to the average of (n=3) biological replicates

| Condition | GG_CO2 | | | GG_N2 | | | GLY_CO2 | | | GLU_CO2 | | |
| --- | --- | --- | --- | --- | --- | --- | --- | --- | --- | --- | --- | --- |
| Experiment | II-A | II-B | II-C | II-A | II-B | II-C | II-A | II-B | II-C | II-A | II-B | II-C |
| Archaea | 5.5±1.3 | 18.1±1.4 | 20.3±2.3 | 3.8 | 19.3 | 9.7 | 14.1 | 8.8 | 18.2 | 16.0 | 13.9 | 23.2 |
| Bacteria | 94.5±1.2 | 81.9±1.4 | 79.7±0.0 | 96.1 | 80.7 | 90.3 | 85.9 | 91.2 | 81.8 | 84.0 | 86.1 | 76.8 |


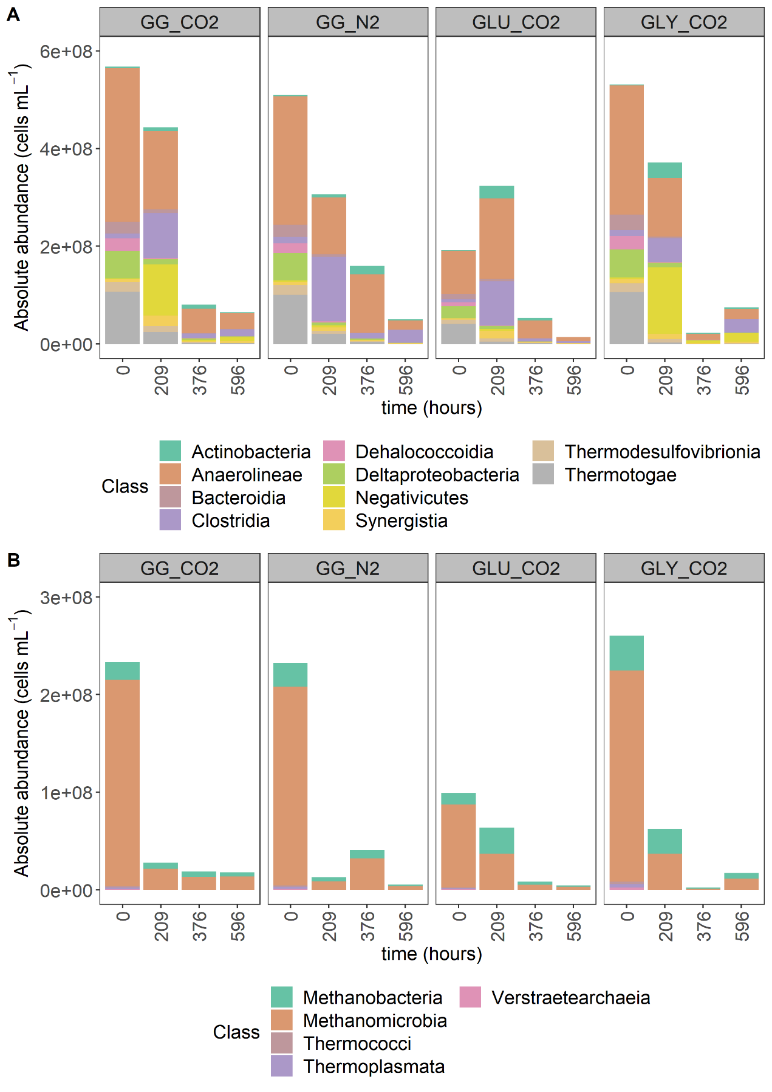


**Figure S1:** Absolute abundances of the top 10 most abundant classes across all treatment and control samples in in A) bacterial community and the top 5 most abundant classes in B) archaeal community. The horizontal axis includes the time points where samples were taken and correspond to the start of experiment II and the end of phase II-A (209 hours), II-B (376 hours) and II-C (596 hours).


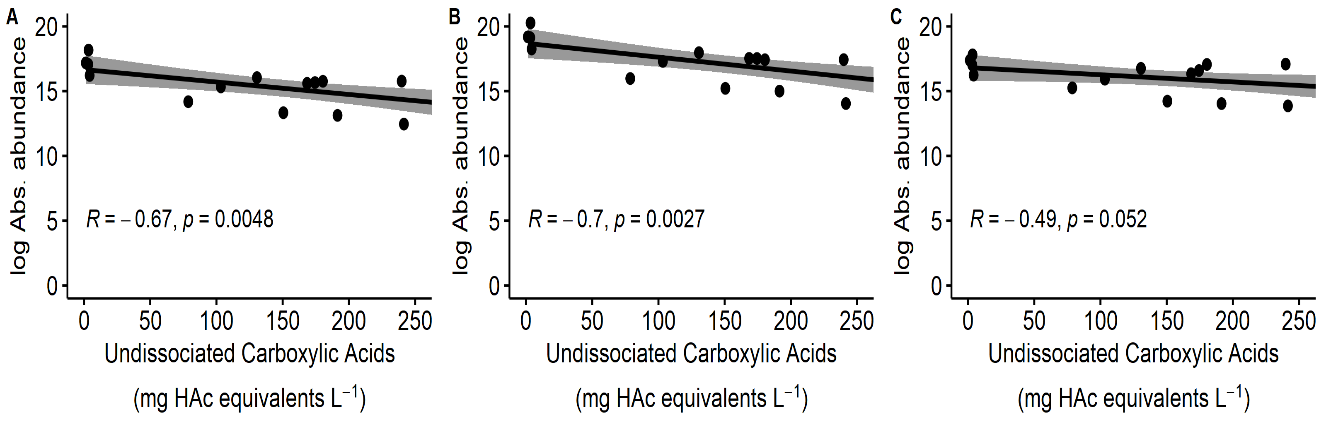


**Figure S2:** Spearman correlation between calculated undissociated carboxylic acids, expressed as mg acetic acid (HAc) equivalents L^-1^, in Experiment II and log-absolute abundances of A) Total Archaea, B) Class Methanomicrobia and C) Class Methanobacteria

**Table S2**: Biochemical reactions possibly involved in glucose and glycerol anaerobic conversion experiment at product and reactant concentrations of 1 mM, initial pH= 7.5 and T=35°C. The Gibbs free energy change (ΔG_cat_^1^) was corrected for pH and calculated according to [9]. Stoichiometries are adapted from [10], [11], [12], [13]

| No. | Catabolic reactions | ΔG_cat_^1^  (kJ/ Rxn) |
| --- | --- | --- |
| 1 | $Glucose \to2 Lactate+2 H^{+}+2 ATP$ | -202.8 |
| 2 | $Glucose \to Butyrate+H^{+}+2 H_{2}+2 CO_{2}+3 ATP$ | -267.0 |
| 3 | $Glucose \to0.67 Acetate+ 0.67 Butyrate+ 1.33 H^{+}+2.67 H_{2}+2 CO_{2}+3.33 ATP$ | -307.8 |
| 4 | $Glucose \to0.67 Acetate+ 1.33 Propionate+ 2 H^{+}+0.67 CO_{2}+2.67 ATP$ | -317.2 |
| 5 | $Glucose+2 Acetate \to2 Butyrate+ 2 CO_{2}+2 H_{2}O+2 ATP$ | -354.3 |
| 6 | $Glucose+0.67 CO_{2}\to1.33 Succinate+0.67 Acetate+0.67 H_{2}O+3.5 H^{+}+2 ATP$ | -292.0 |
| 7 | $Glycerol \to Propionate+H_{2}O+2 ATP$ | -152.5 |
| 8 | $Glycerol \to Lactate+H_{2}+1 ATP$ | -92.4 |
| 9 | $Glycerol \to0.67 Propionate+0.33 Acetate+0.33 CO_{2}+{H_{2}+0.33 H}_{2}O+ 2 H^{+}+1.67 ATP$ | -149.7 |
| 10 | $Glycerol+CO_{2}\to Succinate+H_{2}O+2 ATP$ | -109.3 |
| 11 | $3 Lactate\to Acetate+2 Propionate+2 CO_{2}+1.5 ATP$ | -171.3 |
| 12 | $2 Lactate+ H^{+}\to Butyrate+2 H_{2}+2 CO_{2}+1.5 ATP$ | -64.3 |
| 13 | $Lactate+ Acetate+H^{+}\to Butyrate+CO_{2}+H_{2}O+0.5 ATP$ | -96.9 |
| 14 | $Butyrate+{2H}_{2}O \to2 Acetate+H^{+}+{2H}_{2}+2 ATP$ | +43.9 |
| 15 | $Propionate+{2H}_{2}O \to Acetate+{3H}_{2}+CO_{2}+1 ATP$ | +71.7 |
| 16 | $4H_{2}+2CO_{2}\to Acetate+H^{+}+{2H}_{2}O+1 ATP$ | -55.0 |
| 17 | $4H_{2}+CO_{2}\to CH_{4}+{2H}_{2}O$ | -130.7 |
| 18 | $Acetate+H_{2}O\to CH_{4}+CO_{2}$ | -31.1 |
| 19 | $2 Acetyl-CoA+2 NAD\left( P \right)H \to Butyrate+ATP+2 NAD\left( P \right)^{+}+2 HS-CoA$ | -46.4* |
| 20 | $Acetyl-CoA+H_{2}O \to Acetate+ATP+ HS-CoA$ | -52.1* |

*Values expressed as kJ mol^-1^ acetyl-CoA





**Figure S3:** Gibbs free energy for aceticlastic methanogenesis at circumneutral pH considering the effect of increased partial pressure of carbon dioxide (pCO2 in orange) and acetate concentrations (in green) at T=35°C, pH=7.5 and substrate and product concentrations of 1 mM.

**References**

1. Magoc T, Salzberg SL, Magoč T, Salzberg SL. FLASH: fast length adjustment of short reads to improve genome assemblies. Bioinformatics. 2011;27:2957–63.

2. Bokulich NA, Subramanian S, Faith JJ, Gevers D, Gordon JI, Knight R, et al. Quality-filtering vastly improves diversity estimates from Illumina amplicon sequencing. Nat Methods. 2013;10:57–9.

3. Caporaso JG, Kuczynski J, Stombaugh J, Bittinger K, Bushman FD, Costello EK, et al. QIIME allows analysis of high-throughput community sequencing data. Nat Methods. 2010;7:335–6.

4. Edgar RC, Haas BJ, Clemente JC, Quince C, Knight R. UCHIME improves sensitivity and speed of chimera detection. Bioinformatics. 2011;27:2194–200.

5. Edgar RC. UPARSE: highly accurate OTU sequences from microbial amplicon reads. Nat Methods. 2013;10:996–8.

6. Wang Q, Garrity GM, Tiedje JM, Cole JR. Naive Bayesian Classifier for Rapid Assignment of rRNA Sequences into the New Bacterial Taxonomy. Appl Environ Microbiol. American Society for Microbiology; 2007;73:5261–7.

7. Quast C, Pruesse E, Yilmaz P, Gerken J, Schweer T, Yarza P, et al. The SILVA ribosomal RNA gene database project: improved data processing and web-based tools. Nucleic Acids Res. 2012;41:D590–6.

8. Edgar RC. MUSCLE: multiple sequence alignment with high accuracy and high throughput. Nucleic Acids Res. 2004;32:1792–7.

9. Heijnen JJ, Kleerebezem RR. Bioenergetics of Microbial Growth. Encycl Ind Biotechnol. Hoboken, NJ, USA: John Wiley & Sons, Inc.; 2010. p. 1–66.

10. Fast AG, Papoutsakis ET. Stoichiometric and energetic analyses of non-photosynthetic CO2-fixation pathways to support synthetic biology strategies for production of fuels and chemicals. Curr Opin Chem Eng. 2012;1:380–95.

11. Lee HS, Rittmann BE. Evaluation of metabolism using stoichiometry in fermentative biohydrogen. Biotechnol Bioeng. 2009;102:749–58.

12. Zeng AP, Biebl H, Schlieker H, Deckwer WD. Pathway analysis of glycerol fermentation by Klebsiella pneumoniae: Regulation of reducing equivalent balance and product formation. Enzyme Microb Technol. 1993;15:770–9.

13. González-Cabaleiro R, Ofit¸eru ID, Lema JM, Rodríguez J. Microbial catabolic activities are naturally selected by metabolic energy harvest rate. ISME J. 2016;9:2630–41.
